# The role of low-energy electrons in focused electron beam induced deposition: four case studies of representative precursors

**DOI:** 10.3762/bjnano.6.194

**Published:** 2015-09-16

**Authors:** Rachel M Thorman, Ragesh Kumar T. P., D Howard Fairbrother, Oddur Ingólfsson

**Affiliations:** 1 Science Institute and Department of Chemistry, University of Iceland, Reykjavík, Iceland; 2 Department of Chemistry, Johns Hopkins University, Baltimore, Maryland, USA

**Keywords:** dipolar dissociation, dissociative electron attachment, dissociative ionization, focused electron beam induced deposition (FEBID), low-energy electron-induced fragmentation, neutral dissociation

## Abstract

Focused electron beam induced deposition (FEBID) is a single-step, direct-write nanofabrication technique capable of writing three-dimensional metal-containing nanoscale structures on surfaces using electron-induced reactions of organometallic precursors. Currently FEBID is, however, limited in resolution due to deposition outside the area of the primary electron beam and in metal purity due to incomplete precursor decomposition. Both limitations are likely in part caused by reactions of precursor molecules with low-energy (<100 eV) secondary electrons generated by interactions of the primary beam with the substrate. These low-energy electrons are abundant both inside and outside the area of the primary electron beam and are associated with reactions causing incomplete ligand dissociation from FEBID precursors. As it is not possible to directly study the effects of secondary electrons in situ in FEBID, other means must be used to elucidate their role. In this context, gas phase studies can obtain well-resolved information on low-energy electron-induced reactions with FEBID precursors by studying isolated molecules interacting with single electrons of well-defined energy. In contrast, ultra-high vacuum surface studies on adsorbed precursor molecules can provide information on surface speciation and identify species desorbing from a substrate during electron irradiation under conditions more representative of FEBID. Comparing gas phase and surface science studies allows for insight into the primary deposition mechanisms for individual precursors; ideally, this information can be used to design future FEBID precursors and optimize deposition conditions. In this review, we give a summary of different low-energy electron-induced fragmentation processes that can be initiated by the secondary electrons generated in FEBID, specifically, dissociative electron attachment, dissociative ionization, neutral dissociation, and dipolar dissociation, emphasizing the different nature and energy dependence of each process. We then explore the value of studying these processes through comparative gas phase and surface studies for four commonly-used FEBID precursors: MeCpPtMe_3_, Pt(PF_3_)_4_, Co(CO)_3_NO, and W(CO)_6_. Through these case studies, it is evident that this combination of studies can provide valuable insight into potential mechanisms governing deposit formation in FEBID. Although further experiments and new approaches are needed, these studies are an important stepping-stone toward better understanding the fundamental physics behind the deposition process and establishing design criteria for optimized FEBID precursors.

## Review

### 1 Introduction

**Figure 1:**
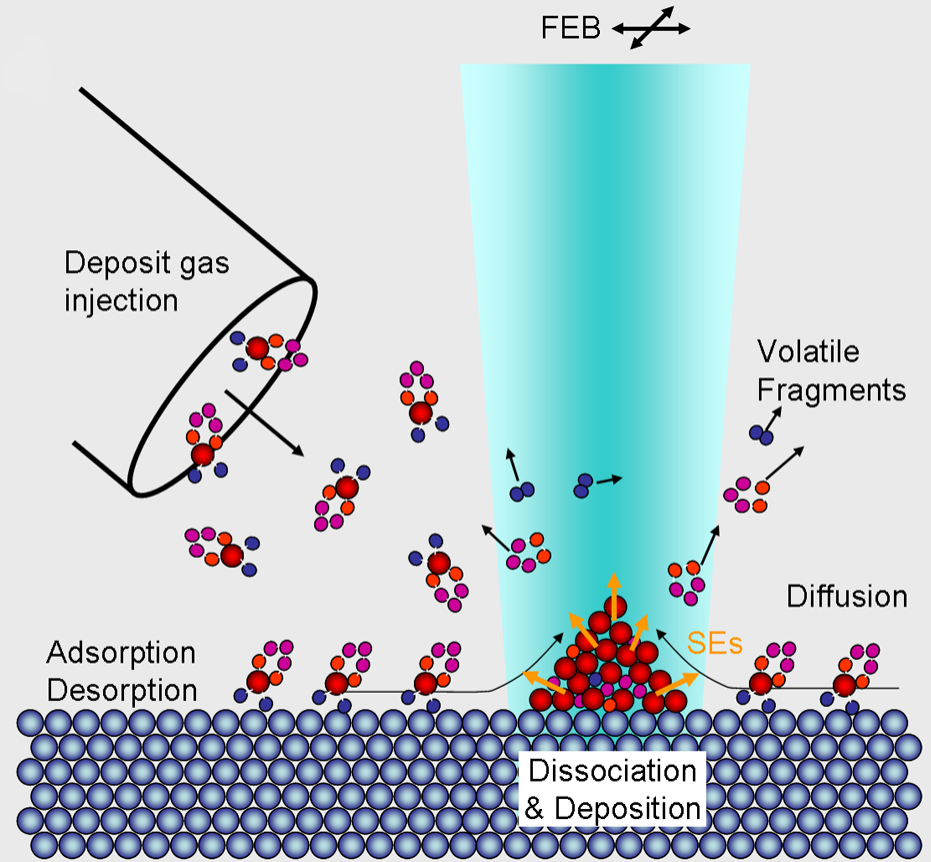
Schematic representation of the FEBID process (reproduced with permission from [2], Copyright (2008) American Vacuum Society): Precursor molecules are supplied through a gas injection system, shown on the left side, and are physisorbed on the surface in dynamic equilibrium with the gas feed. Ideally, the precursor molecules decompose under the electron beam (shown with turquoise shading) to leave a well-defined deposit on the surface, while volatile fragments are pumped away. Diffusion of the physisorbed molecules and the generation of secondary electrons (SEs) are also indicated with black and orange arrows, respectively.

Focused electron beam induced deposition (FEBID) [1–3] is a direct-write method capable of creating nanostructures with potential scientific and industrial applications. The advantages of FEBID stem from its ability to write 3D nanostructures of close to any geometry and to write on uneven surfaces. In FEBID (Figure 1), a focused high-energy electron beam impinges on a surface of a substrate that is continuously exposed to a gas stream of precursor molecules as a material source for the intended deposit. The precursor molecules are physisorbed on the surface in dynamic equilibrium with the gas feed, and ideally decompose under the electron beam to leave a well-defined deposit on the surface. The lateral dimensions of deposited structures are controlled by moving the electron beam and the vertical dimensions are controlled through variation of the dwell time.

Precursor molecules used for depositing metal-containing nanostructures are typically organometallic compounds with a central metal atom and ligand architectures that lend the compounds the following attributes: i) sufficient vapor pressure to facilitate their introduction into a vacuum chamber, ii) chemical stability under ambient conditions and iii) non-toxicity and easy handling. These criteria are the same as those that define suitable precursors for chemical vapor deposition (CVD) [4–5]. Because of this, as well as their widespread commercial availability, FEBID has to-date mainly relied on existing CVD precursors. There is, however, a fundamental difference between the physics and chemistry behind precursor decomposition and deposit formation in CVD and in FEBID. While CVD is primarily thermally driven, FEBID is initiated by electron/molecule interactions. Although thermal effects and surface-induced reactions may also play a significant role in FEBID, the initial electron-driven step will play an important role in defining the final composition of the deposits.

Because the physics and chemistry determining the spatial resolution, aspect ratio, and composition of FEBID deposits is complex, FEBID is unlikely to reach its full capacity through empirical process parameter optimization with currently available CVD precursors. Rather, a sound understanding of the chemistry and physics governing the deposit formation and the translation of such understanding to design parameters for precursor molecules tailored for FEBID is necessary.

**Figure 2:**
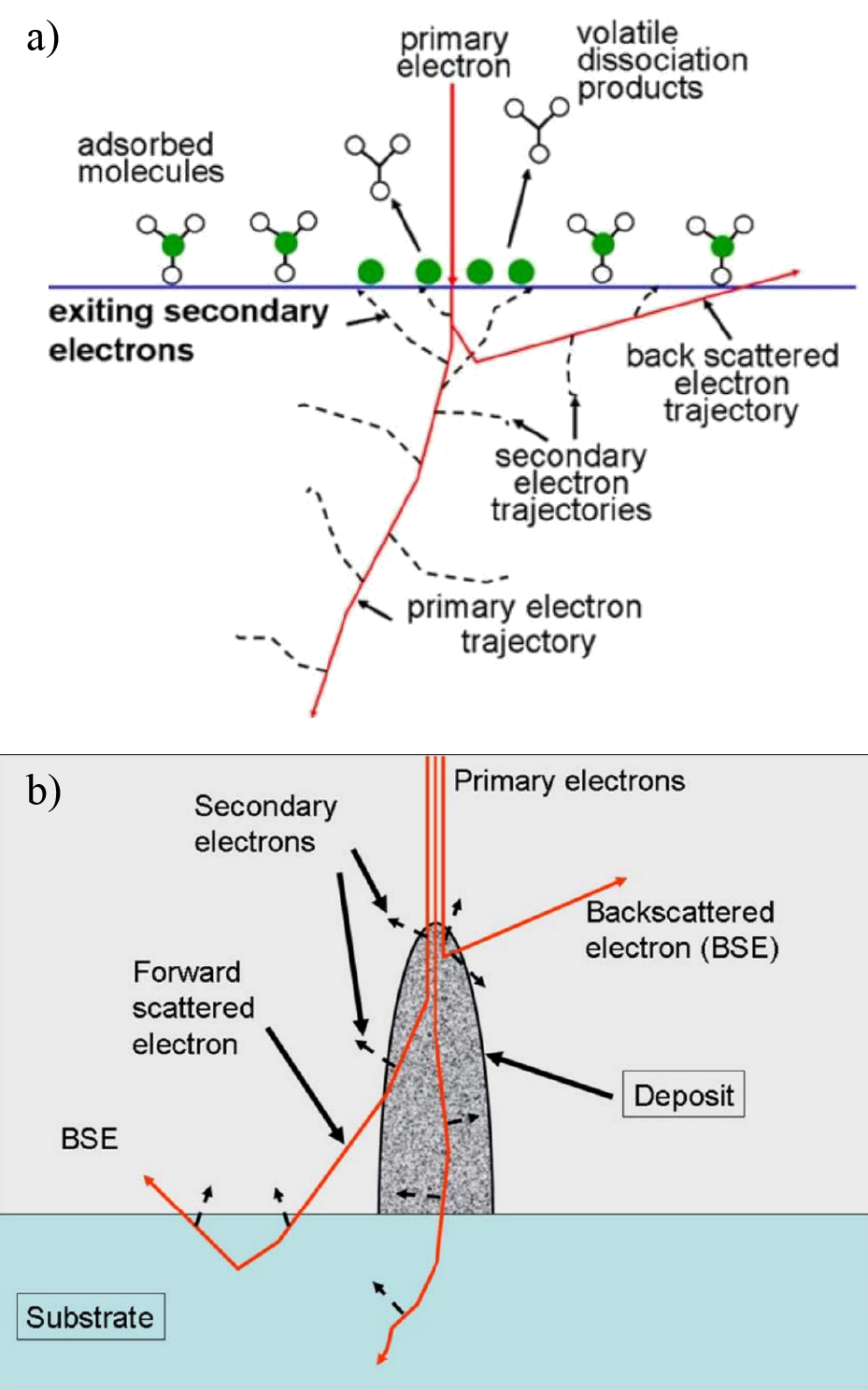
Schematic representation of elastic and inelastic scattering of high-energy primary electrons impinging on a solid substrate and of the generation of SEs through inelastic ionization processes (reproduced with permission from [2], Copyright (2008) American Vacuum Society). (a) Primary electrons impinging on the surface and penetrating the substrate will in part be backscattered and will exit the surface within an area defined by the scattering angle and the electron energy. These electrons lose energy as a result of inelastic, ionizing processes, which in turn, give rise to a large amount of secondary electrons (SEs) produced within the substrate, and more importantly, these SEs are also produced at or close to the surface of the substrate. (b) The same process depicted for a three-dimensional structure growing under the exposure of precursor molecules to the high-energy PE beam. In this case, forward scattered electrons can also reach the surface and produce SEs at the sides of the structure. This may lead to stronger restrictions on the achievable aspect ratios than apply for the achievable resolution of thin layer deposition.

In terms of the electron-induced processes in FEBID, it is clear that the confinement of electron/molecule interactions to the focal width of the incident high-energy electron beam is compromised by elastic and inelastic scattering processes. A portion of the high-energy electrons impinging on the surface and penetrating into the substrate will be backscattered and will exit the surface within an area defined by the scattering angle and the electron energy, rather than by the focal width of the incident beam. Unlike the primary electrons (PEs), which are confined to the focal width of the PE beam, these lower energy scattered electrons will be able to initiate electron-driven reactions outside the area of the PE beam. Moreover, the PEs lose energy as a result of inelastic, ionizing processes which, in turn, give rise to a large amount of secondary electrons (SEs) produced within the substrate. More importantly, these SEs are also produced at or close to the surface of the substrate where they may induce fragmentation of the adsorbed precursor molecules (Figure 2). The reactivity of the SEs with precursor molecules is thus critical in determining the spatial resolution of the deposit. This is even more important with regard to achievable aspect ratios of vertical structures as both backward and forward scattered primary electrons (PEs) and SEs will reach the surface of their sides (Figure 2b).

In general, the SE energy distribution extends with appreciable intensities down to 0 eV, peaks well below 10 eV, and has a higher-energy tail stretching well above 50 eV. The actual form (peak position and width) of the SE *energy distribution* depends largely on the nature of the substrate (work function, Fermi energy, and *Z*-value (atomic number)), and to a lesser extent on the PE energy (as long as it is above about 100 eV) [6–8]. Conversely, the SE *yield* depends significantly on both the nature of the substrate and the PE energy. Note that the former represents the distributions of SE energies while the latter means the total SE yield as function of PE energy. The principal variable determining the influence of the PE energy on the SE yield at the surface is their penetration depth. This, in turn, depends mainly on the *Z*-value of the substrate. In general, the SE yield reaches a distinct maximum well below 1 keV PE energy, before decreasing rapidly again, as is discussed in more detail in context to the commonly used FEBID precursor MeCpPtMe_3_ in section 4.1.

**Figure 3:**
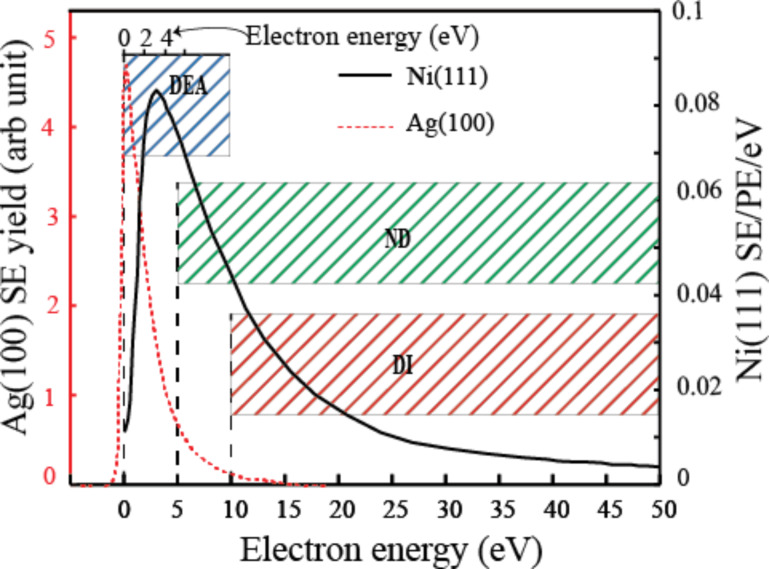
Experimentally measured SE spectra from Ni(111) [6] irradiated by PEs with 400 eV impact energy (black solid line) and from Ag(100) [9] subjected to PEs with 1 keV impact energy (red dotted line). The left-hand *y*-axis shows the relative SE intensity from Ag(100), while the right-hand *y*-axis shows the absolute SE intensity from Ni(111) in SEs per PE per eV (SE/PE/eV). The vertical bars show the approximate electron energy ranges in which the principal electron induced processes are operative, i.e., dissociative electron attachment (DEA), neutral dissociation (ND), and dissociative ionization (DI). The relative extent of these different electron-induced fragmentation processes will depend not only on their relative cross sections, but also on the actual SE energy distribution. From the data shown here, for example, high cross section DEA processes at low energies would likely be dominating for Ag(100) while the integral efficiency of DI and ND processes at higher energies would be more important for Ni(111) (see also [10]).

Figure 3 shows the experimentally determined SE energy distribution for 400 eV PEs impinging on a Ni(111) surface [6] and for 1 keV electrons impinging on a Ag(100) surface [9], along with the approximate electron energy ranges in which the principal electron induced processes are operative, i.e., dissociative electron attachment (DEA), neutral dissociation (ND), and dissociative ionization (DI). While the secondary electron intensity from Ni(111) peaks at about 4 eV with a value close to 0.1 SEs/PE/eV (100 SEs per 1 keV electron) and is still approximately 0.02 SEs/PE/eV at 15 eV [6], the SE intensity from Ag(100) peaks below 1 eV and is already down to 1/10 of the peak intensity at 5 eV [9]. Hence, it is clear that deposit formation in FEBID will be governed by a convolution of the efficiencies of the relevant electron-stimulated processes occurring at the surface and the SE energy distribution at the surface of the substrate. In the case of three-dimensional structures this would be the surface of the growing deposit. Thus, to describe the physics and chemistry of the deposition process in FEBID, the effect of these SEs must be well understood.

This notion that the low energy SEs produced in FEBID may play a significant and even a determining role in the deposit formation has been verified both by simulations [11] and by experiments [12]. In recent years, it has motivated a number of gas phase studies focusing on the energy dependence of the branching ratios and cross sections for various low energy (0–100 eV) electron-induced reactions with organometallic precursors such as Pt(PF_3_)_4_ [13–14], MeCpPtMe_3_ [15], W(CO)_6_ [16–17], Cu(hfac)_2_ and Pd(hfac)_2_ [18], Co(CO)_3_NO [10] and Fe(CO)_5_ [19]. These processes, which are comprised of DEA, DI, ND, and dipolar dissociation (DD), cannot be distinguished in FEBID or surface experiments with high-energy PE beams, where the precursor molecules are simultaneously exposed to a distribution of low energy SEs in addition to the PEs. However, in gas phase experiments, where these precursor molecules interact with well-defined low energy electron beams, the energy dependence and extent of individual fragmentation processes may be unambiguously determined. Such data, in conjunction with surface experiments with high-energy PE beams, may in turn help to understand the mechanism and extent of action of the low energy SEs in the actual FEBID of the same precursor molecules.

In this contribution, we first give a short summary of the different low energy electron-induced fragmentation processes that can occur (DEA, DI, ND, and DD) with emphasis on the different nature and different energy dependence of these processes. We then explore the value of studying these processes through comparative gas phase and surface studies with reference to previously performed gas phase and surface studies of four organometallic FEBID precursors: trimethyl(methylcyclopentadienyl)platinum(IV) (MeCpPtMe_3_) [15,20–21], tetrakis(trifluorophosphine)platinum(0) (Pt(PF_3_)_4_) [13–14,22–23], cobalt tricarbonyl nitrosyl (Co(CO)_3_NO) [10,24–25] and tungsten hexacarbonyl (W(CO)_6_) [16–17,26]. We also discuss these results in the general context of the use of these precursors in FEBID and as part of the ongoing effort to understand the fragmentation mechanisms behind deposit formation. Finally, future perspectives and the relevance of these studies to establishing design criteria for precursor molecules specifically tailored for FEBID will be discussed.

### 2 Low energy electron-induced fragmentation

In the secondary electron energy range relevant for FEBID (<100 eV), there are four distinct mechanisms by which low energy electrons may cause molecular fragmentation, and thus initiate deposition of typical organometallic precursors. These mechanisms are: dissociative electron attachment (DEA), dissociative ionization (DI), neutral dissociation (ND) and dipolar dissociation (DD) [27–32] as depicted in Equations 1–4.

[1]
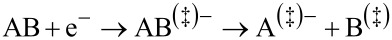


[2]
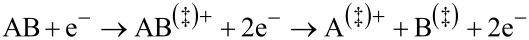


[3]
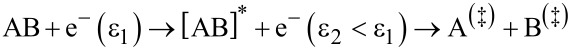


[4]
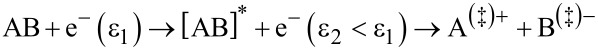


Here; “(‡)” denotes that the fragment(s) may be in a vibrationally and/or electronically excited state, “*” denotes the electronic excitation of the intermediate leading to ND and DD, and ε_1_ and ε_2_ denote the incident energy of the electron and its remaining energy after the inelastic scattering process, respectively.

**Figure 4:**
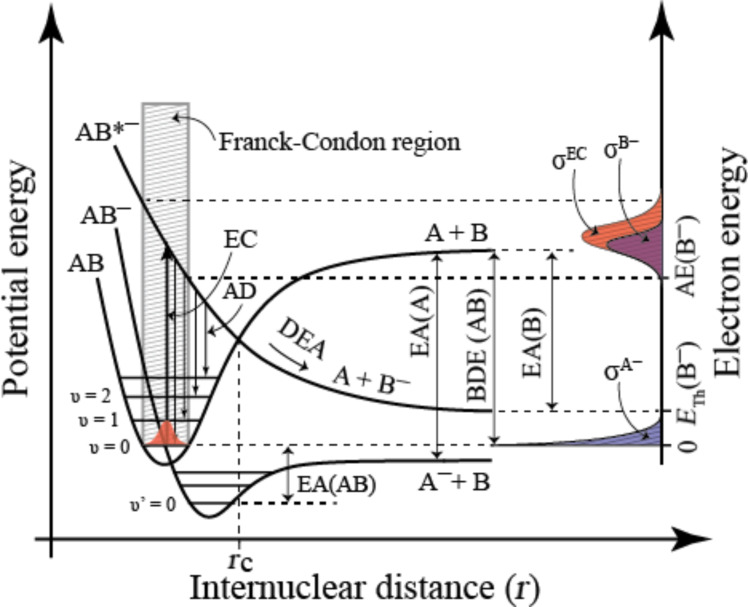
Simplified two-dimensional potential energy diagram for quasi-diatomic dissociation through electron attachment (DEA). The neutral ground state (AB) is depicted along with the anionic ground state (AB^−^) and an electronically excited anionic state (AB*^−^). Electron capture (EC) proceeds through a vertical transition (thick vertical arrow) within the Franck–Condon region (shaded area) and leads to the formation of a transient negative ion (TNI; AB^−^ or AB*^−^ in this case). The TNI formed can then relax through reemission of the electron (autodetachment; AD) which is depicted with thin vertical arrows from AB*^−^ to the neutral electronic ground state. In the case where this reemission process results in a transition to the ground vibrational state, the scattering process is elastic while the other arrows depict vibrationally inelastic processes. The TNI may also relax through nuclear relaxation along the respective anionic potential energy surfaces (DEA). The dissociative asymptotes are here shown to lead to A^−^/B and B^−^/A for AB^−^ and AB*^−^ respectively. For AB^−^ the potential energy curve is shown crossing the vibrational ground state of the neutral; the AB^−^ dissociative asymptote lies below this energy. Hence, the electron affinity of A (EA(A)) is larger than the bond dissociation energy of AB (BDE(AB)). Attachment of a 0 eV electron may thus lead directly to the formation of A^−^. For AB*^−^ a nuclear relaxation beyond the crossing point with the neutral ground state (r_c_) must lead to dissociation, as AD is not possible for nuclear separation beyond this point. The thermochemical threshold for this process (*E*_th_(B^−^)) is given by the difference between the electron affinity of B and the BDE(AB) as depicted on the right-hand y-axis. The appearance energy (AE) for the fragment B^−^, conversely, is defined by the Franck–Condon overlap and is, in this case, substantially higher than *E*_th_(B^−^). This is depicted on the right-hand *y*-axis, in terms of the *reflection principle*, which shows the energy dependence of the electron capture cross section (σ^EC^) as a reflection of the Franck–Condon overlap. The DEA cross section (σ^B−^), which is the product of the attachment cross section and the survival probability of the TNI, i.e., the likelihood that the nuclear relaxation exceeds *r*_c_ before autodetachment is also shown.

**Dissociative electron attachment** (Equation 1) is a resonant process in which an electron is initially captured by the molecule to form a transient negative ion (TNI). This can be understood as a vertical transition from the ground state of the neutral molecule to the ground (or any accessible excited state) of the anion, as is shown in Figure 4. Consequently, the TNI formed is generally in a vibrationally and/or electronically excited state. Under collision-free conditions, it relaxes rapidly either through re-emission of the electron (autodetachment; AD) or through dissociation (DEA). Dissociative electron attachment is active below the ionization threshold of the molecule and generally most efficient at very low incidents energies. The cross section for a given DEA process is defined by the initial attachment cross section multiplied by the probability that the TNI survives nuclear relaxation beyond the crossing point of the respective potential energy curves (*r*_c_ in Figure 4). Thus, DEA is confined to narrow energy ranges defined by the Franck–Condon overlap of the wave function of neutral ground state and the respective negative ion states, and by the survival probability of the TNI with regard to AD. At very low electron energies where s-wave attachment dominates, the cross section is proportional to *E *^−1/2^ [33] (see also [34]) and the cross section is thus highest at threshold (i.e., close to 0 eV). The survival probability is also high close to the threshold as the distance to the crossing point of the anionic ground state is short (as is depicted in Figure 4). Such a threshold process is depicted for the lower anionic potential curve in Figure 4, which is shown crossing the ground vibrational state of the neutral molecule, favoring transitions at or close to 0 eV incident electron energy (see also the caption to Figure 4).

For FEBID, the consequence of DEA being most efficient close to 0 eV incident electron energy is that this process is only likely to contribute significantly to precursor decomposition at very low incident electron energies. Moreover, as dissociation will generally proceed along the initial anionic potential energy surface, selective single bond ruptures dominate in DEA. For such a process to be thermochemically accessible at 0 eV, the electron affinity of the neutral corresponding to the anionic fragment formed (A in Equation 1) must exceed the bond dissociation energy (BDE) of the bond being broken (A–B in Equation 1).

It should, however, be noted that the treatment here is simplified to a quasi-diatomic model and molecular rearrangement and formation of new bonds upon electron capture can in some cases lead to considerably more fragmentation with fairly high cross sections at low incident energies. Good examples of such reactions are the extensive fragmentation of tetrafluorophenol and tetrafluoroaniline [35] as well as that of the commonly used FEBID precursor ligands tri- and hexafluoroacetylacetone [36]. In each of these cases, low energy electron attachment leads to the formation of neutral HF, which in turn releases the 5.9 eV HF BDE [37] and promotes further fragmentation of the parent molecule. This is also observed for other molecules such as the amino acids glycine [38] and valine [39], and hexafluoroacetone azine [40], wherein the formation of molecular hydrogen and ethane enables the otherwise thermochemically inhibited formation of CN^−^ at low incident electron energies. With a suitable choice of ligands, such intramolecular reactions may thus also provide a new means to enhance fragmentation of potential FEBID precursors through DEA.

Perhaps more important in FEBID is the fact that interaction of precursor molecules with the surface of the substrate may alter the DEA cross sections substantially. This may be simply due to the enabled energy transfer offering a new relaxation path that competes with DEA (and AD). Conversely, in other instances polarization interactions may stabilize the TNI with respect to autodetachment and facilitate DEA [41–42].

**Figure 5:**
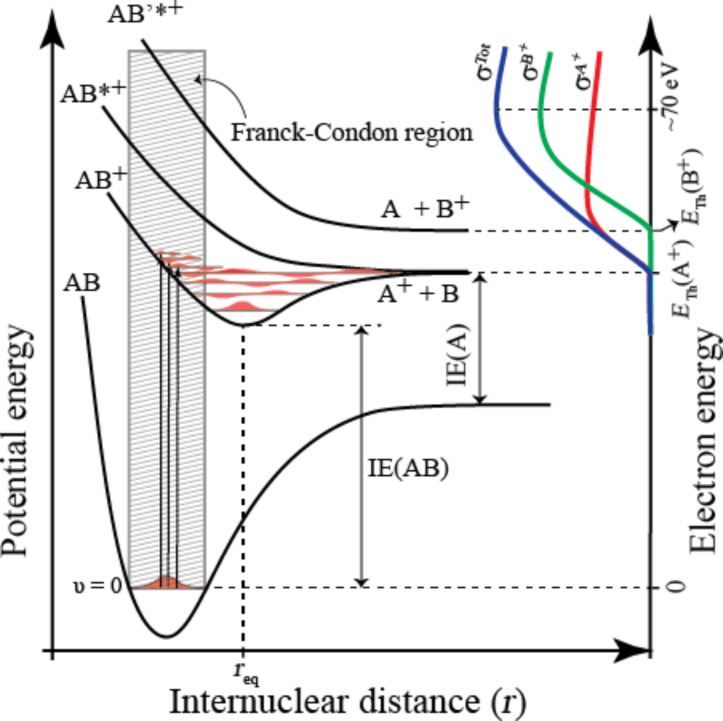
Simplified two-dimensional potential energy diagram for a quasi-diatomic dissociation through electron impact ionization (DI). The ionization process is depicted as a vertical transition from the neutral ground state to the cationic ground state and higher lying cationic states within the Franck–Condon region (shaded area). In this representation the cationic ground state has a considerably larger equilibrium bond length (*r*_eq_) compared to the neutral, leading to a significant transition probability to vibrational states that are energetically above the dissociation limit. For simplification, the excited cationic states (AB^+^* and AB^+^′*) are shown to be purely repulsive. In this representation the parent cation is formed as long as the electron incident energy is above the ionization energy of AB (IE(AB)) but below the dissociation threshold leading to the formation of A^+^ (*E*_Th_(A^+^). The higher-lying excited cationic state (AB^+^′*) is shown to lead to the formation of B^+^. The threshold for this channel is given by the sum of the ionization energy of B (IE(B)) and the bond dissociation energy of AB (BDE(AB)). On the right-hand *y*-axis, the energy dependence of the relative cross section for the formation of A^+^ and B^+^ (σ ^A+^ and σ ^B+^) are shown as red and green lines and the total DI cross section (σ^Tot^) is shown with a solid line as the sum of the two partial cross sections. The threshold energy (*E*_Th_) for the formation of the fragments A^+^ and B^+^ are indicated on the right-hand *y*-axis and the maximum total DI cross section is shown to be at 70 eV. In the case of a polyatomic molecule, the situation is considerably more complex and intramolecular energy re-distribution, multiple fragmentations and rearrangement reactions may dominate the ion formation at higher energies.

**Dissociative ionization** (Equation 2) is fundamentally different from DEA. Here, energy transfer from the incident electron leads to removal of a bound electron from the target molecule and the formation of a parent cation. Similarly to DEA, this can be depicted as a vertical transition of an initially bound electron to the ionization continuum of the molecule as shown in Figure 5. However, if the incident energy in the electron/molecule collision exceeds the ionization energy of the respective molecule, part of the “excess” energy can be transferred to the molecule. This will leave the parent cation in a vibrationally and/or electronically excited state, which often leads to fragmentation. In this case, the extent of the fragmentation and the branching ratios between different fragmentation channels depends on the internal energy of the ion and the thermochemical threshold (or activation energies) for the respective processes. The onset for DI in terms of electron energy is therefore generally slightly above the ionization energy of the molecule and is initially defined by a single bond rupture. With increasing incident electron energy, however, the branching ratios shift more and more to favor multiple bond ruptures, while the total cross section approaches a maximum (typically at around 50–70 eV) before decreasing slowly again. At higher incident electron energies, the interaction time is shorter and the scattering cross section (and thus the DI cross section) decreases again (see pages 23–25 in [43]).

The consequence for FEBID is that DI is likely to contribute to more extensive fragmentation of the precursor molecules when compared to DEA, and moreover that DI will typically only contribute through precursor interaction with the high-energy tail of the SE energy distribution (above about 10 eV). Since DI is a non-resonant process, the total cross section remains fairly constant over a large energy range above the respective thresholds. This will often result in a substantial integral overlap with the SE energy distribution. Hence, while DEA can only proceed through resonances confined to narrow energy ranges below about 10 eV, DI is active from slightly above the molecule’s ionization energy to well above 100 eV.

**Figure 6:**
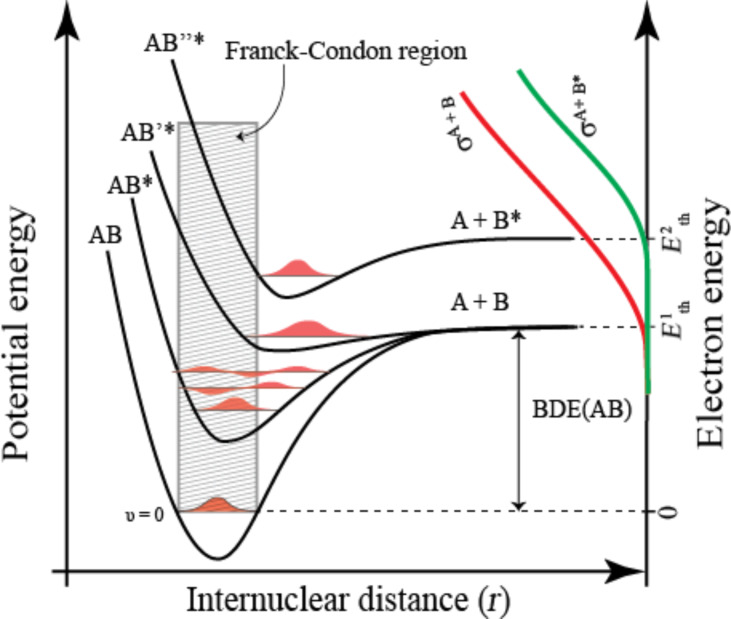
Simplified two-dimensional potential energy diagram for a quasi-diatomic dissociation through electronic excitation, i.e., neutral dissociation (ND). The excitation process is depicted as a vertical transition from the neutral ground state to electronically excited states accessible within the Franck–Condon region (shaded area). Here, the two lower-lying excited states AB^*^ and AB′^*^ are shown as bound states and dissociation can only proceed through transitions to vibrationally excited states that are energetically above the respective dissociation limit. In this representation, both AB^*^ and AB′^*^ dissociate to form A and B in their respective ground states. The highest-lying excited state (AB″^*^), on the other hand, is shown to be purely repulsive and to dissociate directly to form A and B^*^. On the right-hand *y*-axis, the energy dependence of the relative cross section for the formation of A and B and A and B* (σ^A+B^ and σ^A+B*^ ) are shown as red and green lines and the total cross section for ND (σ^Tot^ ) is shown with a solid line as the sum of the two partial cross sections. The threshold energy (*E*_Th_) for the respective processes is indicated on the right-hand *y*-axis. Similarly to DI and DEA, the situation in ND is more complex in the case of a polyatomic molecule, and intramolecular energy redistribution, multiple fragmentations, and rearrangement reactions may play an important role.

**Neutral dissociation through electronic excitation** (Equation 3) has characteristics of both DEA and DI. As depicted in Figure 6, it shows a threshold behavior similar to DI, as the initial electronic excitation energy defines the threshold for the process (if it is higher than the respective BDEs). The cross section for individual processes then gradually increases as the electron energy increases and more higher-lying excitation channels open up, also contributing to the total cross section. Unlike DI, the energy transfer is largely confined to the electronic excitation energy, though the resulting electronic states may generally be expected to be vibrationally excited. The available energy is thus limited by the energy characterizing the respective electronic transition in the molecule and the excess vibrational energy associated with the transition. Neutral dissociation is therefore not expected to lead to as extensive fragmentation as DI. However, as the first excited states in organometallic compounds may be as low as 3–4 eV and the ligand BDE is usually low compared to covalent bonds, ND may be active at much lower energies than DI. Similar to DI, ND may still maintain fairly high cross sections for incident electron energies, even in excess of 100 eV (depending on the energy transfer efficiency). Furthermore, electronic excitation from bonding orbitals to strongly anti-bonding orbitals can result in direct dissociation along the respective repulsive potential energy surface, similarly to DEA. In fact, recent quantum mechanical calculations on the potential energy surfaces of selected electronically excited states of Pt(PF_3_)_4_ show the repulsive nature of these states along the metal–ligand bond [44]. However, although these calculations are a considerable achievement, they do not predict whether the remaining internal energy leads to further fragmentation or is channeled into kinetic energy of the departing fragments. Also, in a recent dissociative excitation study on Fe(CO)_5_ [19], measurements on the incident electron energy dependence of the induced fluorescence of the fragments formed were attributed to ND processes leading to partial and even complete CO loss up on electron impact. However, as is the case for the studies on Pt(PF_3_)_4_ these studies do not give measures of the efficiency of these processes.

**Dipolar dissociation** (Equation 4) proceeds similarly to ND, but the Coulomb interaction between the negatively and positively charged fragments must be overcome. The thermochemical threshold for this process is given by the sum of the respective BDEs and the ionization energy of the precursor of the positive ion formed less the electron affinity of the precursor of the negative ion formed. The threshold is thus generally higher than that for DEA and ND but lower than that for DI. To our knowledge there are no current gas phase studies on DD of relevant FEBID precursors and generally DD is not a very efficient process (see T. D. Märk and references therein on pages 276–277 in [27]) It is, however, worth mentioning that a recent study on electron-stimulated negative ion desorption from Fe(CO)_5_ films shows a significant contribution to the desorption yield from dipolar dissociation [45].

In addition to the different energy dependence of these electron-stimulated processes and the different extent of fragmentation, it should be emphasized that the fragmentation paths will also be distinctly different. While DEA predominantly leads to the formation of a closed shell anion and a neutral, radical counterpart, DI will predominantly result in a closed shell cation and neutral radical counterparts and ND in neutral radical fragments. Thus, one would expect that that the relative importance of these processes in FEBIP will not only define the initial step in the deposition process, but may also strongly influence further surface, thermal, or electron-induced chemical transformation of the deposit.

### 3 Gas phase vs surface studies

To study low energy electron-induced processes in the gas phase, a low energy electron beam with a resolution of about 100 meV is crossed with an effusive beam of FEBID precursor molecules and the electron energy dependence for the formation of charged fragments is monitored by mass spectrometry (MS) with sufficient resolution and dynamic range to unambiguously detect all fragments formed. For experimental detail relevant to the DEA and DI data discussed here, see [46–48]. Using this methodology, an accurate assessment of the branching ratios for individual DEA and DI fragmentation channels may be achieved and, with careful calibration, absolute cross sections may be determined. Such instruments may also be used to determine the extent of DD, but the extent of ND must currently be estimated from scattering experiments measuring the cross sections for the underlying electronic excitations. For experimental detail on the determination of the scattering cross sections discussed here, see [49]. Regardless of the experimental apparatus or the electron-stimulated processes being investigated, all of these gas phase experiments study single electron/molecule collision events for isolated species and thus do not necessarily reflect the low energy electron-induced decomposition pathways of the same molecules when adsorbed onto a substrate. This may be addressed with well-controlled UHV surface experiments where the precursors are adsorbed onto a substrate and exposed to electrons with relatively high energy (400–500 eV). In such surface experiments, the desorbing fragments are analyzed with MS and the composition of the remaining deposit can be analyzed with techniques such as X-ray photoelectron spectroscopy (XPS), reflection-absorption IR spectroscopy (RAIRS), and/or high-resolution electron energy loss spectroscopy (HREELS). For experimental details on the UHV surface experiments discussed here, see [25,50]. The shortcoming of these surface experiments is, however, that the precursor molecules are subjected to interactions with SEs with an energy distribution (similar to that in FEBID), in contrast to the well-defined incident energies that characterize the gas phase studies. The energy dependence of the observed processes is therefore not known directly. Consequently, a comparison of the products formed in gas phase and surface science studies combined with the energy dependence of the branching ratios (i.e., the products) obtained in gas phase experiments is needed to identify the dominant processes (e.g., DEA vs DI) occurring in the surface reactions. Such comparison provides valuable insight into the dominant low energy electron-induced processes occurring with FEBID precursors adsorbed on surfaces and may eventually aid the formulation of distinct criteria defining suitable ligand structure and composition for future FEBID precursors.

### 4 Case studies

#### 4.1 Trimethyl(methylcyclopentadienyl)platinum(IV); MeCpPtMe_3_

The organometallic compound trimethyl(methylcyclopentadienyl)platinum(IV) (MeCpPtMe_3_) was first tested as a CVD precursor by Xue et al. [51] in 1989 and was found to create high-purity platinum films, with purities greater than 99 atom % platinum when examined by XPS. Despite its high deposit purity using thermal deposition techniques, MeCpPtMe_3_ has not been found to produce high-purity deposits in direct FEBID and such deposits do not exceed about 20 atom % Pt [52–54]. In this context, Botman et al. [54] examined FEBID-constructed platinum structures deposited at various surface power densities, calculated from the deposition beam voltage and current and the SE escape area of the substrate. The platinum purity of the unprocessed deposit was optimized to a maximum of approximately 16 atom % at power densities at and above 10 µW/µm^2^, but substantially reduced platinum purities were observed at lower power densities (as low as 5.5 atom %). With thermal and electron beam-assisted in situ and post-deposition treatment with processing gases, however, considerably higher Pt content has been achieved [55–56], and resistivity only about six times that of bulk Pt may be attained [57–58]. Such approaches include exposure to atomic hydrogen [54], water [59–60] and oxygen [57–58,61–62], but also the combination of FEBID with atomic layer deposition [63] and with laser exposure [64] have proven advantageous. Despite the poor purity of Pt deposits created from MeCpPtMe_3_ in the absence of any purification strategies, MeCpPtMe_3_ has continued to be used as a FEBID precursor due to its stability, good vapor pressure under FEBID conditions, and commercial availability.

A 2012 study by S. Engmann et al. [15] deals with the gas phase dissociation of MeCpPtMe_3_ upon exposure to low-energy electrons. Gas phase experiments were performed using a crossed electron beam/effusive molecular beam apparatus and product ions were measured using mass spectrometry; the apparatus and methods have each been described in detail [46]. As previously described, electrons with incident energies below the ionization threshold of the parent molecule (7.7 eV for MeCpPtMe_3_ [15]) can only produce ionic fragments via DEA (DD usually sets in at higher energies). Hence, negative ions collected from electron/molecule interactions at such low incident electron energies are DEA products.

**Figure 7:**
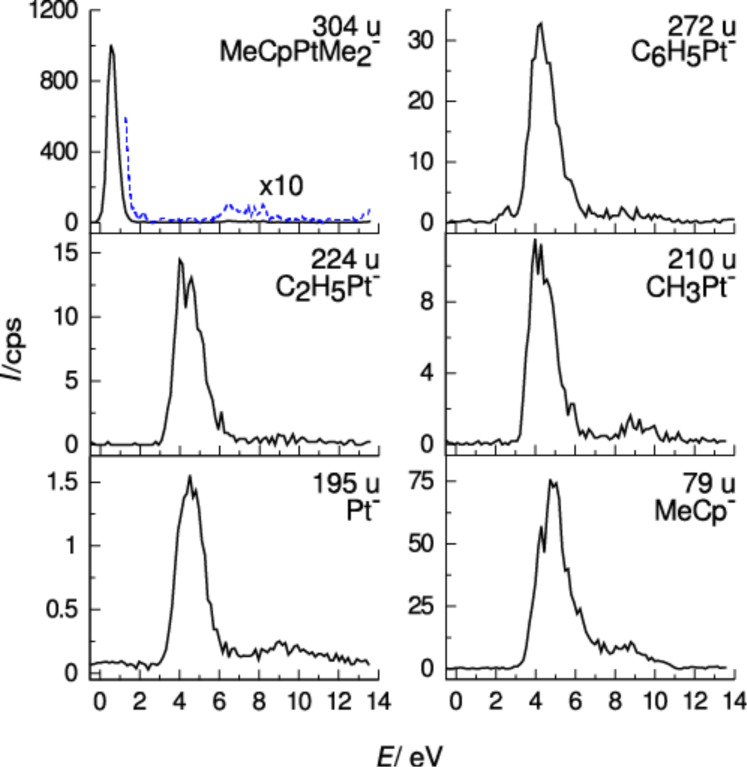
Energy-dependent relative cross sections (ion yields) of negative ion fragments produced by DEA to MeCpPtMe_3_. Reproduced with permission from [15], Copyright (2012) Royal Society of Chemistry.

Figure 7 shows the negative ion yields from DEA to MeCpPtMe_3_ in the incident electron energy range of 0–14 eV. The highest intensity DEA fragment is at *m*/*z* 304 and results from a single methyl loss (CH_3_), yielding the [MeCpPtMe_2_]^−^ ion. This fragment is almost exclusively produced through a low energy resonance, which the authors assigned to a single electron occupation of the LUMO of MeCpPtMe_3_. This is anticipated to be predominantly antibonding along the Pt–CH_3_ coordinate [65–66]. The onset of the [MeCpPtMe_2_]^−^ formation in the DEA ion yield curves is close to 0 eV, and the peak intensity of this fragment is close to 0.5 eV. Multiple ligand loss through DEA, on the other hand, proceeds predominantly through a higher energy resonance, which the authors assigned as a core-excited resonance (two-particle-one-hole resonance) associated with a HOMO–LUMO transition. The fragment formation through this resonance peaks close to 4.5 eV. From thermochemical considerations it is clear that multiple ligand loss through DEA is in all cases accompanied by significant rearrangement and new bond formation. This can be seen particularly well in the case of the [C_7_H_11_Pt]^−^ fragment (*m*/*z* 290), which is the only multiple ligand loss fragment formed through the low energy resonance close to 0 eV (though with very low intensity). For the formation of this fragment, the authors proposed a reaction pathway involving the elimination of an ethyl radical via the intramolecular attack of a leaving methyl radical on another methyl ligand and a H-shift from one of the methyl ligands to the central Pt, thereby reducing the central Pt(IV) to Pt(II) [15]. Such extensive rearrangement reactions have, to our knowledge, not been observed for DEA to other organometallic compounds, but have been observed for a number of covalently bonded compounds as discussed in the previous section. In addition to the resonances discussed above, all fragments observed also appear at higher energies through broad low intensity contributions.

Finally, [C_7_H_9_Pt]^−^ (*m*/*z* 288) is observed through a fairly narrow contribution peaking at 2.3 eV [15] (not shown here). This fragment may be attributed to a loss of an ethyl radical and H_2_ or, alternately, to the loss of an ethane molecule and a hydrogen radical, and is assigned as a single particle shape resonance. The ratio of the highest-intensity DEA fragment, [MeCpPtMe_2_]^−^ to the next highest fragment, [MeCp]^−^ (*m*/*z* 79) (leaving a neutral fragment with a maximum C/Pt ratio of 3:1), is approximately 13:1, and all other fragments resulting from multiple ligand loss appear with even lower intensity. Hence, single methyl loss dominates in DEA of MeCpPtMe_3_.

**Figure 8:**
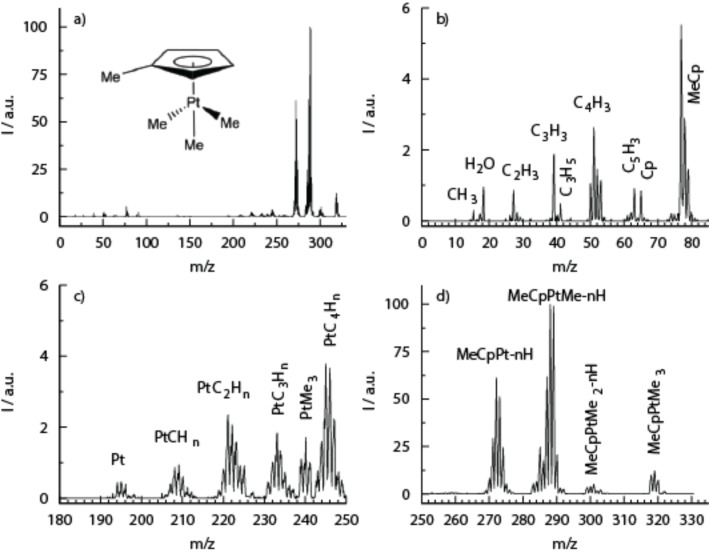
Positive ion mass spectrum of MeCpPtMe_3_ recorded with electron energy of 100 eV (reproduced with permission from [15], Copyright (2012) Royal Society of Chemistry); (a) overview of the full mass range (*m*/*z* 0–330), (b), (c) and (d); expanded portion of the spectrum for *m*/*z* 0–85, *m*/*z* 180–250 and *m*/*z* 250–330, respectively.

Figure 8 shows the positive ion mass spectrum of MeCpPtMe_3_ recorded at 100 eV incident electron energy, where DI dominates [15]. The principal DI channels are the loss of two or three methyl ligands and the loss of two or three methyl ligands along with one or more hydrogen. The loss of one methyl group (and one methyl group and one or more hydrogen) is about an order of magnitude less efficient, and all other fragmentation channels are even less efficient. The DI fragmentation patterns are complicated by the presence of isobaric fragments generated through hydrogen loss – specifically, through the overlap of fragments with different platinum isotopes and those with differing extent of hydrogen loss. Thus, a determination of the threshold energy for individual fragments was not possible, but it is safe to assume that the relative ratios observed at 100 eV impact energy represent fairly well the ratios over the bulk of the relevant SE energy distribution in FEBID.

From this comparison of gas phase DEA and DI data on MeCpPtMe_3_, it is evident that the most efficient DEA channel is the loss of one methyl ligand. In contrast, the highest intensity DI channel is the loss of two methyl groups, along with the loss of two methyl groups and one or more hydrogen atoms. The second most efficient DI channel is half as intense and corresponds to the loss of three methyl ligands along with the loss of three methyl ligands and one or more hydrogen.

Assuming that MeCpPtMe_3_ adsorbed on a surface will react similarly to the gas phase, DEA to MeCpPtMe_3_ adsorbed on a surface should lead to a reduction of the C/Pt ratio from the initial 9:1 in the intact molecule to close to 8:1, while DI of MeCpPtMe_3_ adsorbed on a surface should lead to a deposition with C/Pt ratio between 7:1 and 6:1.

**Figure 9:**
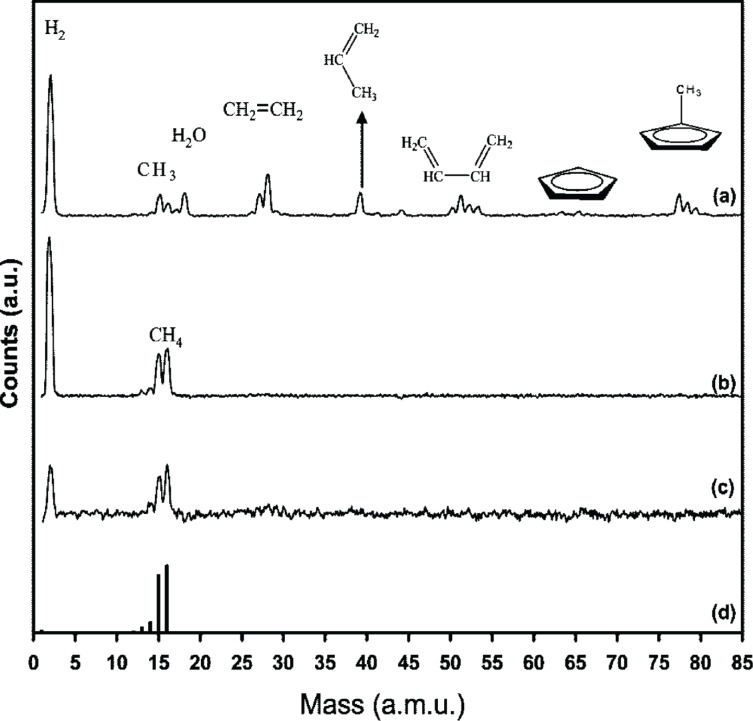
Positive ion mass spectra of MeCpPtMe_3_ in the *m*/*z* range of 0–85 (reproduced with permission from Wnuk et al. [21], Copyright (2009) American Chemical Society): (a) gas phase spectrum recorded at 70 eV impact energy; (b) positive ion mass spectrum of volatile species desorbing from a MeCpPtMe_3_ film, adsorbed onto a gold substrate at 180 K, under irradiation with 500 eV electrons; (c) positive ion mass spectrum of volatile species desorbing from a CpPtMe_3_ film, adsorbed onto a gold substrate at 180 K, under irradiation with 500 eV electrons; (d) reference mass spectra of gas phase CH_4_ (adapted from NIST [67]).

A 2009 paper by J. D. Wnuk et al. [21] describes such surface experiments performed using MeCpPtMe_3_. These experiments were performed using UHV chambers equipped with XPS and MS, and with RAIRS, respectively, as well as commercial flood guns for use as electron sources. The MeCpPtMe_3_ precursor was physisorbed onto gold substrates at about 180 K and irradiated with electrons with 500 eV impact energy. During electron irradiation, a MS with a 70 eV electron impact ionization source was used to monitor desorption of volatile decomposition products from the surface, while XPS and RAIRS were used to monitor the evolution of the composition of the forming deposit. Figure 9 shows the mass spectrum of species desorbing from the surface before and during electron irradiation.

The only visible species during electron irradiation are at *m*/*z* 15, 16, and 2. These are assigned to CH_3_^+^, CH_4_^+^, and H_2_^+^, with the former two appearing at the ratio observed in electron impact ionization of gas phase methane (CH_4_). The initial loss of a methyl radical is likely to result from a Pt–CH_3_ bond rupture rather than by dissociation of the methyl group from the MeCp ligand, as the BDE for the latter is expected to be more than 2 eV higher than for the former [15]. This was confirmed by Wnuk et al. [21] through a supplementary study of the analogue cyclopentadienyltrimethylplatinum(IV) (CpPtMe_3_), which produced a similar mass spectrum during electron irradiation, despite the lack of the methyl group on the Cp ring. The conversion of the dissociated methyl radicals to the methane observed in the mass spectra is less clear as it could arise either from intra- or intermolecular reactions at the surface or from reactions of desorbed methyl radicals at the walls of the UHV chamber. While MeCpPtMe_3_ was found to desorb from the surface when it was heated to room temperature prior to electron irradiation, no compounds were found to desorb from the surface after electron irradiation. Hence, through electron irradiation, a chemical change clearly converted the physisorbed MeCpPtMe_3_ to a chemically bound deposit containing platinum and carbon.

**Figure 10:**
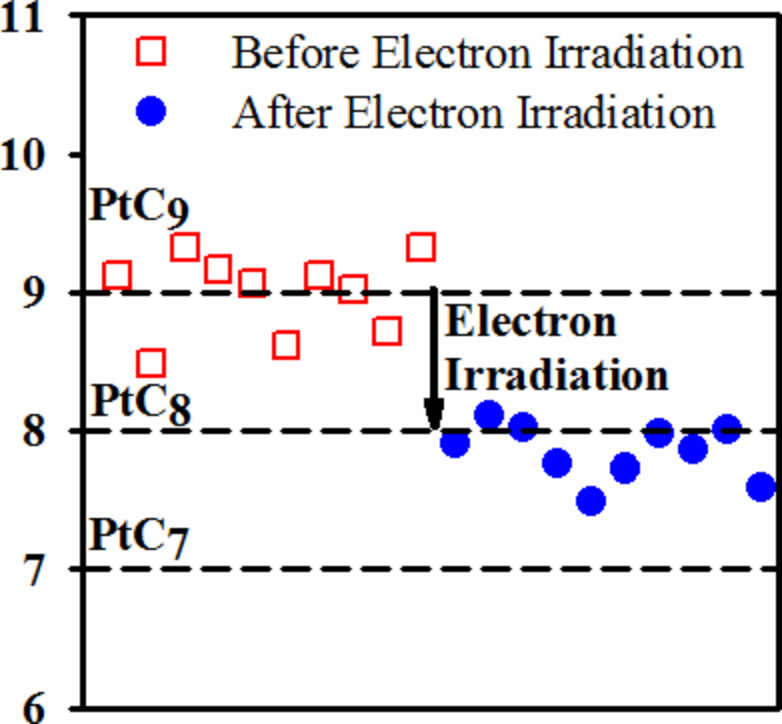
Changes in the C/Pt ratio of a 3.16 nm thick film of MeCpPtMe_3_ adsorbed on a gold surface at 180 K (reproduced with permission from Wnuk et al. [21], Copyright (2009) American Chemical Society). The open squares show the C/Pt ratio before electron irradiation and filled circles show the C/Pt ratio after irradiation with 500 eV electrons. The dashed horizontal lines indicate the expected C/Pt ratio for films with stoichiometry of PtC_9_, PtC_8_ and PtC_7_.

In addition to the mass spectra recorded to monitor desorbing fragments, the evolution of the surface composition with increasing electron dose was monitored by XPS and RAIRS. The XPS spectra showed that the fractional Pt coverage stayed constant, but a partial reduction of Pt(IV) to a lower oxidation state took place. The C/Pt ratio decreased from the initial 9:1 of the precursor molecule to about 8:1 upon electron irradiation, as is shown in Figure 10. This ratio was found to remain the same for initial film thicknesses of 1–3 nm and for incident electron irradiation with 500 and 200 eV electron energy. Furthermore, the RAIRS data showed a systematic loss of absorbance in the ν(C–H) stretching region with increasing electron dose. The authors interpreted their findings as due to an initial electron-induced Pt–CH_3_ bond rupture caused by DEA, which was initiated by low energy secondary electrons.

This is consistent with the expected single ligand loss through DEA, as observed in the gas phase experiments. Moreover, considering the currently available gas phase data, this implies that electron-induced decomposition of surface-adsorbed MeCpPtMe_3_ is predominantly caused by secondary electrons with incident energy below 1 eV.

**Figure 11:**
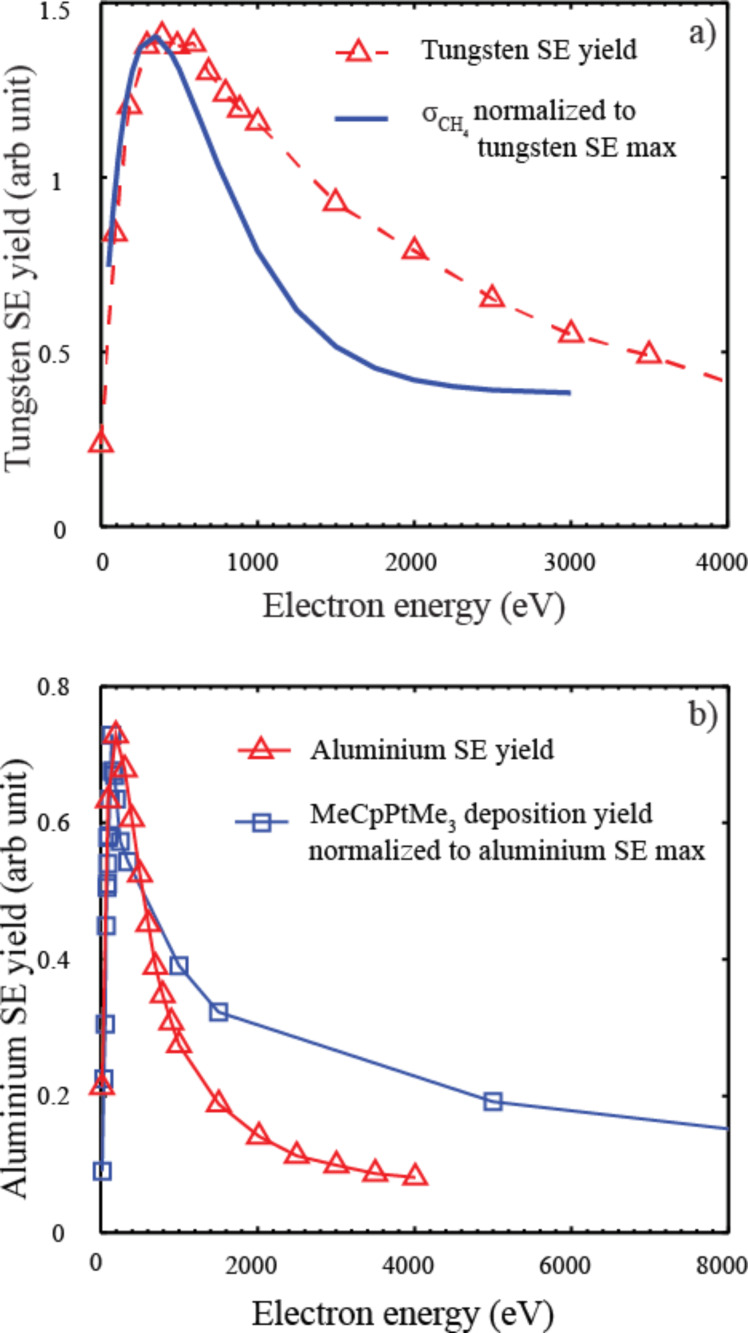
a) A line of best fit to the cross section for methane desorption from MeCpPtMe_3_ adsorbed on a gold surface (*Z* = 97) and exposed to electron irradiation in the energy range from 0 to about 3000 eV (solid blue line), compared to energy dependence of the SE yield of tungsten (*Z* = 94) as a function of primary electron energy (red triangles). b) The deposition yield of MeCpPtMe_3_ on a silicon surface (*Z* = 14) as a function of the PE beam energy in the range from about 0–5000 eV (blue squares) compared to the energy dependence of the SE yield from aluminum (*Z* = 13) as a function of primary electron energy (red triangles). The methane desorption cross sections (a) are adapted from Wnuk et al. [68] and the deposition yield (b) is adapted from Botman et al. [12]. The calculated SE yields are adapted from Ohya et al. [8] (using the partial wave expansion for the cross-section). Lines connecting the symbols are only meant to guide the eye.

Interestingly, the cross section for methane desorption from adsorbed MeCpPtMe_3_ exhibits a qualitatively similar PE energy dependence as that expected for the SE yield. This is also true for the MeCpPtMe_3_ deposition yield as function of incident electron energy, and may be taken as further support for the notation that the low energy SEs are driving the deposition. To demonstrate this, Figure 11a compares a best fit to the cross section for methane desorption from MeCpPtMe_3_ physisorbed on a gold surface (*Z* = 79) and the calculated PE energy dependence of the SE yield from tungsten (*Z* =74). The comparison with tungsten is chosen as its atomic number, which strongly influences the PE energy dependence of the SE yield, is close to that of gold. Further, Figure 11b compares the PE energy dependence of the MeCpPtMe_3_ deposition yield on a silicon surface (*Z* = 14) and the calculated PE energy dependence of the SE yield from aluminum (*Z* = 13). Here, one has to keep in mind the influence of the growing deposit on the effective *Z*-number in the measurements of the deposition yield, as this may be closer to that of carbon. In both cases (Figure 11a and Figure 11b) the calculated energy dependence is adapted from Ohya et al. [8]. The scatter in the cross section data for the methane desorption is considerable and the same is true for the errors in the deposition cross section. Furthermore, the exact peak position and the general form of the energy dependence of the calculated SE yield depend strongly on the model used and the *Z*-number of the respective substrate material, with the work function and Fermi level of the substrate also playing a role. Nevertheless, the qualitative similarities between the energy dependence of the measured cross sections and that of the calculated SE yields is evident and clearly supports the notation that the role of the SEs is dominating in the deposit formation.

#### 4.2 Tetrakis(trifluorophosphine)platinum(0) Pt(PF_3_)_4_

Tetrakis (trifluorophosphine) platinum (0) (Pt(PF_3_)_4_), is a Pt-containing FEBID precursor that does no contain carbon; this has the potential advantage that unlike MeCpPtMe_3_, it cannot create carbon-contaminated deposits. It is liquid at ambient temperatures, has a vapor pressure of 65 Torr, and is fairly stable at room temperature when stored under a PF_3_ atmosphere [69–70]. It has been shown to produce pure Pt deposits using CVD [70]. In FEBID, deposits with a Pt content as high as 81 atom % have been achieved [71]. This is a considerably higher Pt content than achieved by FEBID of MeCpPtMe_3_, where deposits typically contain less than 20% Pt [52–54]. Post-deposition procedures have also been studied and found to further improve the percent platinum content and conductivity. In this context, a platinum content of about 94 atom % has been attained through annealing in the presence of H_2_O [72] and a resistance of 0.24 × 10^−3^ Ω·cm (only an order of magnitude higher than the bulk value for Pt) has been reached through annealing under nitrogen and a mixture of nitrogen with 5% hydrogen [73]. Tetrakis(trifluorophosphine)platinum is also one of the best-studied FEBID precursor with regards to the molecular mechanisms behind its deposition. These studies include absolute cross section measurements for DEA of Pt(PF_3_)_4_ [14] and a determination of the thermal electron attachment rate constant and the associated activation energy using a flowing-afterglow Langmuir probe [74] as well as absolute cross section measurements for elastic, vibrational, and electronic scattering [13]. Collectively, these studies provide insight into electron energy loss processes that occur through interaction with the precursor and internal excitation of the precursor, and the potential role of ND in the deposit formation. All of these gas phase studies can be compared with a UHV surface study by Landheer et al. [23], where mass spectrometry was used to monitor desorption of volatile decomposition products and XPS and HREELS were used to monitor the evolution of the deposit during irradiation.

**Figure 12:**
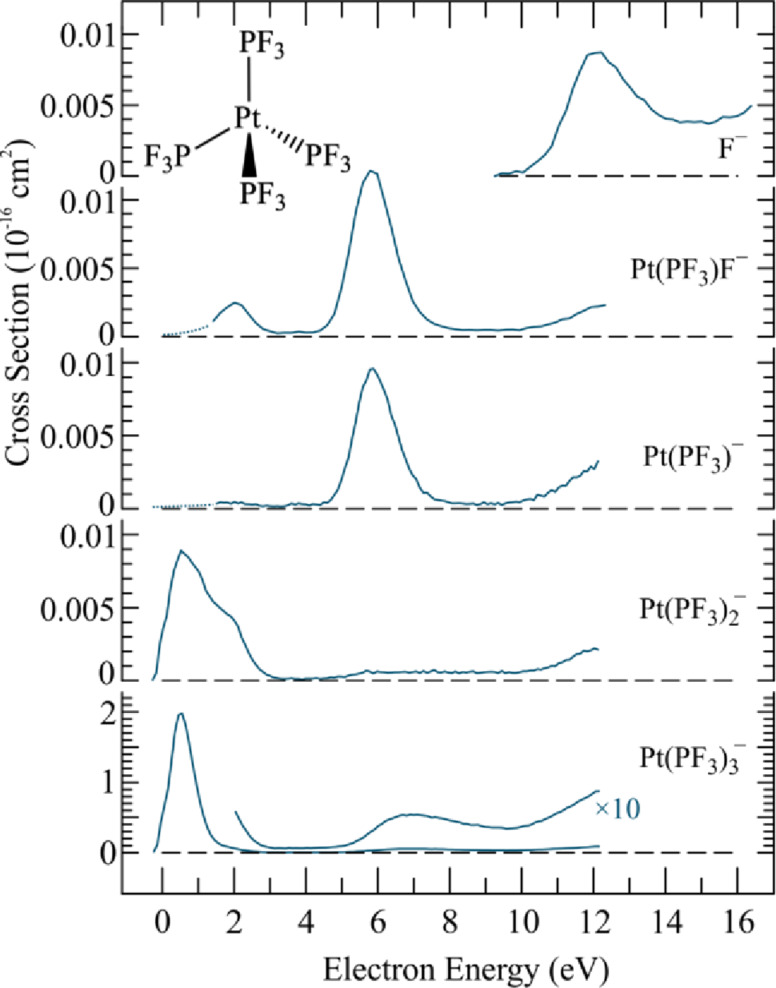
Energy-dependent absolute cross sections for negative ion fragments produced by DEA to Pt(PF_3_)_4_ (reproduced with permission from May et al. [14], Copyright (2012) Royal Society of Chemistry).

**Figure 13:**
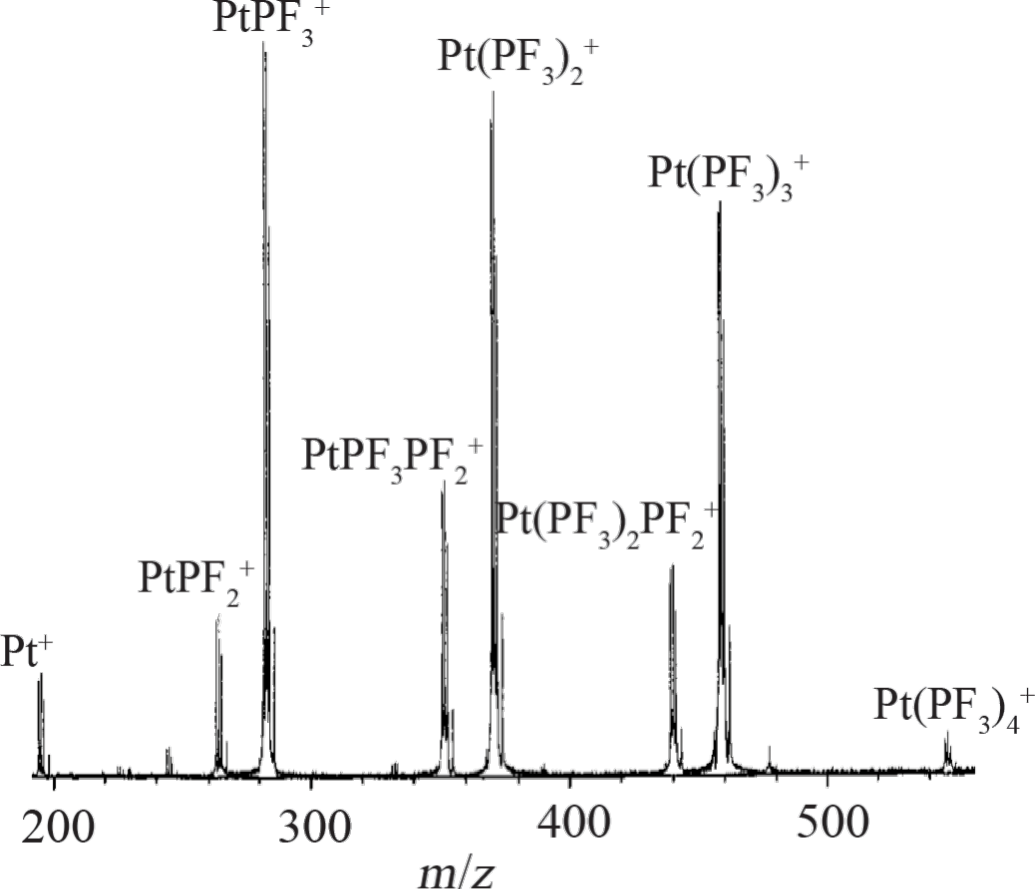
Electron ionization FT-ICR mass spectrum of Pt(PF_3_)_4_ recorded at 20 eV incident electron energy (reproduced with permission from [22], Copyright (1997) American Chemical Society).

Figure 12 shows the energy dependence of the DEA cross sections for Pt(PF_3_)_4_ in the energy range from 0–12 eV and Figure 13 shows a positive ion FT-ICR mass spectrum of Pt(PF_3_)_4_ ionized with an axial beam of 20 eV electrons. The DEA spectra are dominated by single ligand (PF_3_) loss peaking at about 0.5 eV with the very high cross section of 2 × 10^−16^ cm^2^. This value is only about an order of magnitude lower than the maximum theoretical cross section for s-wave attachment at this energy [27]. All other channels are about two orders of magnitude less efficient; from these, the loss of two PF_3_ ligands also proceeds through the resonance appearing close to 0.5 eV in the DEA spectra. Further fragmentation leading to the formation of [Pt(PF_3_)]^−^ and [Pt(PF_3_)F]^−^ proceeds predominantly through a higher lying resonance appearing close to 6 eV in the DEA ion yield, and F^−^ appears through a broad contribution close to 12 eV.

Unfortunately no quantitative data on the energy dependence of DI is available for Pt(PF_3_)_4_, but the FT-ICR spectrum [22] allows for qualitative comparison (see Figure 13).

It is clear from this spectrum that even at 20 eV electron impact energy, DI leads to considerably more extensive fragmentation than DEA. While single ligand loss is dominant in DEA, the relative cross sections for the loss of one, two, and three ligands in DI at 20 eV are all comparable. Additionally, both the loss of one fluorine atom in addition to the respective ligands (e.g., Pt(PF_3_)_2_PF_2_), and the formation of the bare platinum cation contribute appreciably to the total DI yield. For higher electron impact energies, it is safe to assume that the DI branching ratios will shift further to favor more extensive fragmentation. This is a general behavior, an example of which can be seen clearly for the energy dependence of DI of W(CO)_6_ [17] and Co(CO)_3_NO [10], which are discussed hereafter. Thus, DEA to Pt(PF_3_)_4_ leads predominantly to single ligand loss while DI integrated over the energy range of the SEs generated in FEBID will predominantly lead to more extensive fragmentation.

**Figure 14:**
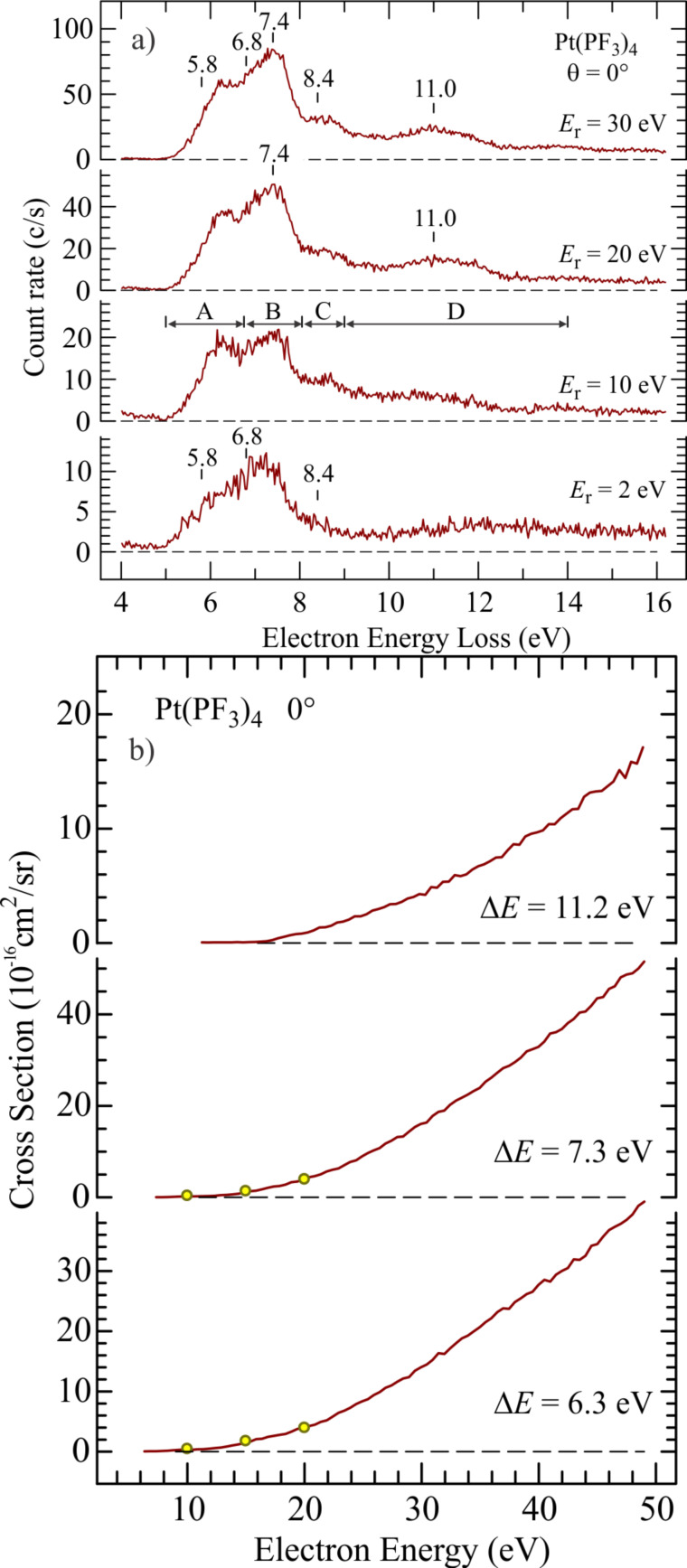
(a) Electron energy loss spectra of Pt(PF_3_)_4_ recorded at 0° angle with varying residual energies. The sections marked A–D in the third panel represent the energy ranges in which integration is performed to obtain absolute values. (b) Absolute electron excitation cross sections as a function of electron energy integrated over the energy loss ranges signified as A, B and D in Figure 14a. These cross sections are recorded at 0° angle and electron energy losses of 6.3 eV, 7.3 eV and 11.2 eV. Both figures (a) and (b) are reproduced with permission from [13], Copyright (2011) American Institute of Physics.

In addition to the absolute DEA cross sections shown in Figure 12, Allan [13] has determined absolute cross sections for the angular dependence and energy dependence of elastic, vibrational, and electronic scattering of electrons from Pt(PF_3_)_4_ (mainly focused on the region below 20 eV incident electron energy). While elastic scattering will influence the spatial distribution of the secondary electrons and vibrational scattering will contribute to heating of the precursor molecules and substrate, electronic excitations may lead to direct ND, and therefore potentially play a significant role in the initial step in the FEBID process. Figure 14a,b compares the electron energy loss spectra for Pt(PF_3_)_4_ for different residual electron energies (Figure 14a) and the incident electron energy dependence of the absolute cross sections for electronic excitation (Figure 14b) integrated over the energy ranges signified as A, B, and D in the third panel of Figure 14a. The data is recorded at 0° scattering angle. It is clear that the electronic excitations in Pt(PF_3_)_4_ have cross sections comparable to those measured for DEA. Unfortunately, no conclusive data exists on the extent and nature of the dissociation processes resulting from these electronic excitations. From the mass spectra reported by Hammill et al. [22] one can, however, speculate on the energetics involved and, therefore, on the possible extent of neutral fragmentation from the individual electronic excitations. It can be seen from Figure 12 that the bare Pt cation is produced from Pt(PF_3_)_4_ at 20 eV electron impact energy. Since the ionization energy of Pt is 9 eV [75], the 11 eV of residual energy must be sufficient to effect the loss of all four PF_3_ ligands, indicating that the average Pt–PF_3_ ligand bond dissociation energy is less than 2.75 eV. Thus the loss of two ligands may already be energetically accessible through the bulk of the excitation region marked A in Figure 14a, and three or even all four ligands may be lost through ND via the higher lying electronic excitations.

Recent quantum mechanical calculations on these states show that the potential surfaces of the four lowest electronically excited states are dissociative with respect to a single ligand loss, similarly to the DEA process [44]. However, as is apparent from the calculations above, the remaining internal energy may lead to further dissociation.

**Figure 15:**
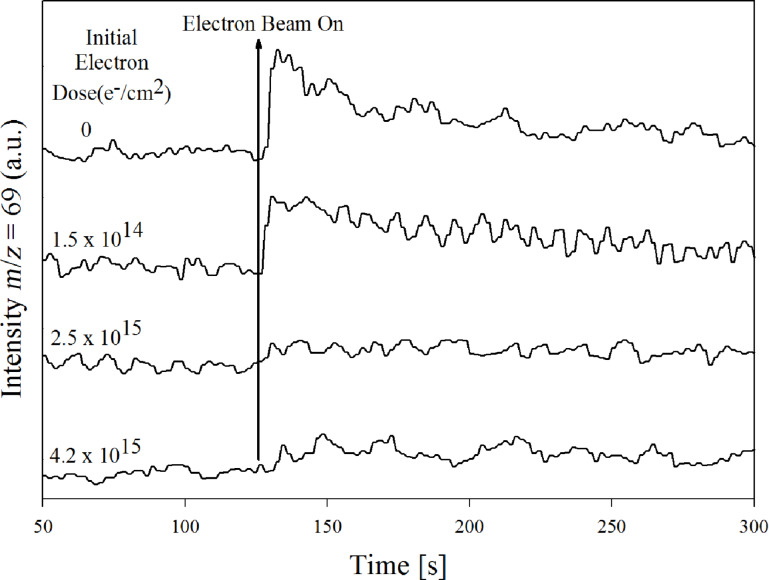
Time-resolved mass spectra of gas phase PF_3_ (positive [PF_2_]^+^ ions produced by 70 eV electron impact are recorded) desorbing from films of Pt(PF_3_)_4_ adsorbed on an Au surface at 180 K when these are irradiated with 500 eV electrons. The time at which the electron beam is turned on is represented with a vertical line. Reproduced with permission from [23], Copyright (2011) American Chemical Society.

Under UHV conditions, Landheer et al. [23] have studied the decomposition of Pt(PF_3_)_4_ molecules, adsorbed at low temperatures (<170 K), upon irradiation with 500 eV electrons. In these experiments, neutral fragments desorbing from the substrate during electron irradiation were monitored by mass spectrometry, and while the chemical composition of the remaining film was probed by XPS, HREELS was used to study the change in vibrational modes of the adsorbed molecules. Figure 15 shows a time-resolved mass spectrum monitoring the PF_3_ desorption during electron exposure after an initial electron dose of 0, 1.5 × 10^14^, 2.5 × 10^15^, and 4.2 × 10^14^ e^−^/cm^2^.

**Figure 16:**
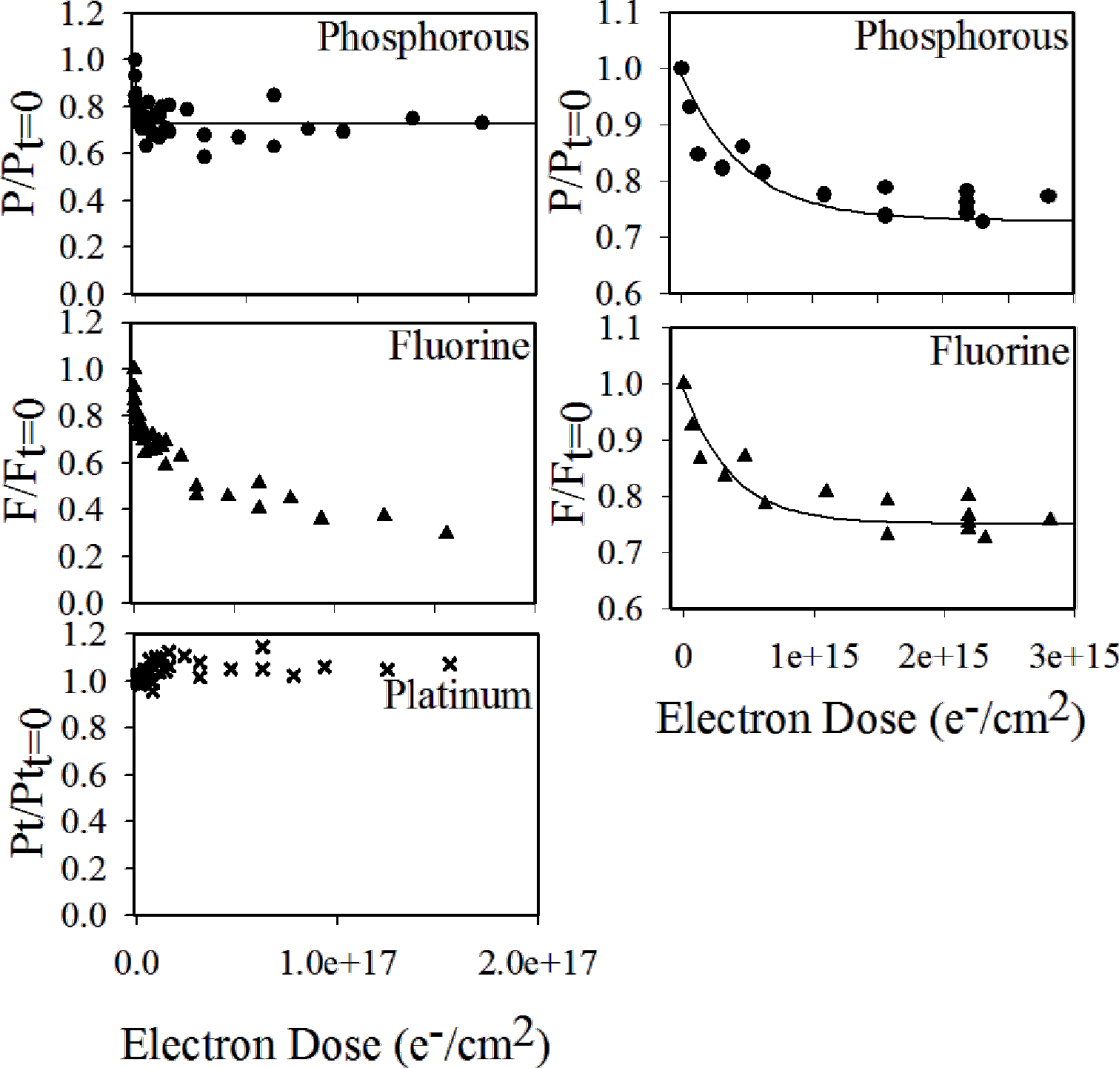
Electron dose dependence of the fractional coverage of Phosphorous (P/P_D=0_), Fluorine (F/F_D=0_) and Platinum (Pt/Pt_D=0_). The left-hand side plots show the changes in fractional coverage for the full range of electron doses up to 2 × 10^17^ e^−^/cm^2^. The right-hand side plots show the phosphorous and fluorine fractional coverage for the initial period of electron exposure up to 2 × 10^15^ e^−^/cm^2^. Solid lines in the plot show a fit to experimental values based on the first order loss process. Reproduced with permission from [23], Copyright (2011) American Chemical Society.

Figure 16 shows the electron dose dependence of the fractional platinum, phosphorous, and fluorine coverage determined by XPS [23]. The mass spectrum clearly shows an electron-initiated PF_3_ desorption, and that this desorption comes to a halt at an electron dose slightly above 10^15^ e^−^/cm^2^. The loss of phosphorous and fluorine in the same electron dose range is also apparent from the XPS data, which shows that both the fractional phosphorous and fluorine contents decrease rapidly, and at the same rate as one another, to 75% of their initial 1:4 ratio. Electron exposures above 3 × 10^15^ e^−^/cm^2^ resulted in additional fluorine loss and oxygen deposition, while the fractional phosphorous content remained stable at 75% of its initial value [23]. The authors interpreted these observations as a two-step process. In the first step, electron interaction with Pt(PF_3_)_4_ leads to a single Pt−PF_3_ bond rupture and transformation of the adsorbed Pt(PF_3_)_4_ to Pt(PF_3_)_3_. This explains why the phosphorous and fluorine signals decrease at the same rate to 75% of their initial values while the P/F ratio remains constant. In the second step, further electron exposure nearly exclusively leads to P–F bond rupture and the coordinately unsaturated phosphorous reacts with residual water to form phosphorous oxides.

If we compare the evidence of single PF_3_ ligand loss as the initial step in the deposit formation (as shown by the XPS data) with the existing experimental gas phase data, the observed single ligand loss points strongly towards DEA, rather than DI, as the initiator of deposition. This is not conclusive, however, as the role of ND is not certain without further information on the relaxation of the respective electronically excited states. Interestingly, if DEA is in fact the dominating process, the electron-induced deposition of Pt(PF_3_)_4_ is initiated by SEs with an incident electron energy of less than 1 eV, and the rest of the SE energy distribution as well as the primary electrons play an insignificant part in this primary step. Conversely, if ND in fact plays an important role in the initial single ligand loss, then energies above about 10 eV will also be important as is apparent in Figure 14. This notion, however, presumes that the processes observed in the gas phase remain similar with regards to the fragmentation, when Pt(PF_3_)_4_ molecules are adsorbed on surfaces.

#### 4.3 Cobalt tricarbonyl nitrosyl; [Co(CO)_3_NO] and tungsten hexacarbonyl [W(CO)_6_]

Cobalt tricarbonyl nitrosyl [Co(CO)_3_NO] was initially introduced in CVD as a liquid, easy-to-handle Co source. [76–78]. In CVD, Crawford et al. [78] reported an average composition of CoN_0.5_O_0.9_ with only traces of carbon when using Ar or N_2_ as carrier a gas at deposition temperatures below 380 °C. Above 400 °C, the authors found the deposits to consist of a mixture of CoO and Co metal. Deposition under hydrogen atmosphere, on the other hand, was found to result in pure cobalt deposits already at 350 °C [76–77]. Cobalt tricarbonyl nitrosyl has a normal boiling point of 78.6 °C, a vapor pressure of 91 Torr at 20 °C [79], and a thermal decomposition temperature of about 130–140 °C measured on SiO_2_ [80]. It is also commercially available and relatively nontoxic. Furthermore, the commonly used Co precursor Co_2_(CO)_8_ is unstable under vacuum and tends to polymerize, releasing CO. This, in turn, may lead to pressure buildup in precursor reservoirs, complicating the protocol for its use [81]. Consequently, a number of FEBID studies and an electron beam-induced surface activation (EBISA) study have been conducted on Co(CO)_3_NO [80–83]. In these studies, at room temperature, the Co content of the deposits was found to be about 40–50 atom %, independent of beam energy and current. The Co/N and Co/C ratios are similar in these deposits (about 3.5:1), while the initial ratios in the precursor molecule are 1:1 and 1:3, respectively. Hence, carbon loss is clearly much more pronounced than nitrogen loss. Furthermore, EDX and TEM studies along with resistivity measurements indicate that the deposit consists of Co nano-grains embedded in an insulating CoO matrix [82]. The chemical speciation of the nitrogen and carbon in the deposits remains an open question in these studies. However, as the bulk of the oxygen is bound as CoO, the nitrogen is likely bound as the respective cobalt nitride.

Focused electron beam induced deposition of Co(CO)_3_NO at elevated substrate temperatures [80] leads to a substantial decrease in the carbon content (by about a factor of three). At 50 °C, the oxygen content decreases fairly abruptly to about 50% of its initial value (from 15 to about 7.5 atom %); at 100 °C, the nitrogen content increases by approximately the same atomic percentage. The cobalt content, gradually increases from about 40 atom % at room temperature to about 50 atom % at 200 °C. This results in a composition of approximately CoC_0.15_O_0.45_N_0.45_. We are not aware of studies on post-deposition or in situ purification of the deposits formed with Co(CO)_3_NO, but in a recent study, the combination of annealing, H_2_ exposure, and electron irradiation of deposits formed with Co_2_(CO)_8_ was found to result in compact, carbon and oxygen free Co layers [84].

**Figure 17:**
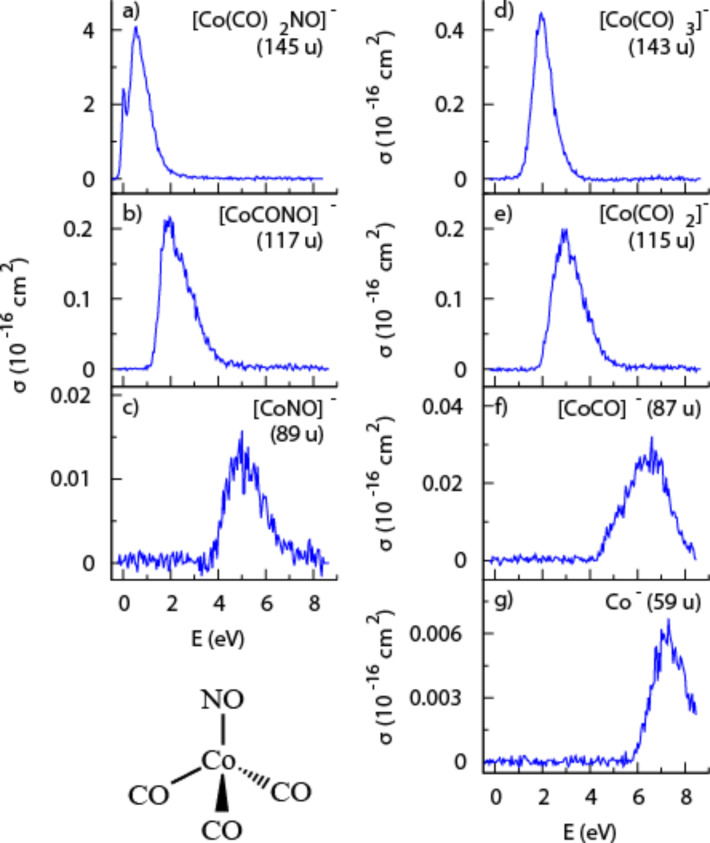
Energy-dependent absolute DEA cross sections for Co(CO)_3_NO (reproduced with permission from [10], Copyright (2013) American Institute of Physics).

In a 2011 gas phase study, Engmann et al. [24] published absolute cross section values for DEA to Co(CO)_3_NO. These were the first absolute cross section values published for DEA to any potential FEBID precursor. The energy dependence of these cross sections is shown in Figure 17.

Similar to MeCpPtMe_3_ and Pt(PF_3_)_4_, the most efficient channel in DEA to Co(CO)_3_NO is a single ligand loss close to 0 eV. Moreover, the absolute cross section for single ligand loss through DEA is very high. For Co(CO)_3_NO, the loss of one CO ligand was assigned to the formation of a single particle resonance resulting in a maximum [Co(CO)_2_NO]^−^ yield close to 1 eV incident energy, while hot band transitions associated with the same resonance result in another maximum close to 0 eV. The maximum cross section for [Co(CO)_2_NO]^−^ formation was found to be about 4 × 10^−16^ cm^2^ and, although the uncertainty associated with these measurements is considerably larger than these associated with the measurements by May et al. for Pt(PF_3_)_4_ [14], these cross sections are clearly very high. The loss of the NO ligand is also observed but is confined to the energy range from about 1–3 eV with a peak intensity close to 2 eV and a maximum cross-section that is an order of magnitude lower than that for the single CO loss. This NO loss channel was assigned as a low-lying two-particle-one-hole resonance associated with a HOMO–LUMO transition. The loss of two or more ligands, i.e., the formation of [Co(CO)NO]^−^, [Co(CO)_2_]^−^, [CoNO]^−^, and [CoCO]^−^, is observed in the range from about 2 eV to about 6 eV and is attributed to further decomposition of [Co(CO)_2_NO]^−^ and [Co(CO)_3_]^−^ at the high energy tail of the respective resonances, where there is sufficient energy to induce further fragmentation. However, the maximum cross section for the formation of these fragments in this energy range is only about 5% of the cross section for single CO loss for [Co(CO)NO]^−^ and [Co(CO)_2_]^−^, and about 0.5% for [CoNO]^−^ and [CoCO]^−^. [Co(CO)]^–^ is also formed through a higher-lying core-excited resonance but with a maximum cross section close to 0.5% of that for single CO loss, while the bare Co^−^ ion is also formed through the same resonance with a cross section that is close to 0.2% of that for single CO loss.

**Figure 18:**
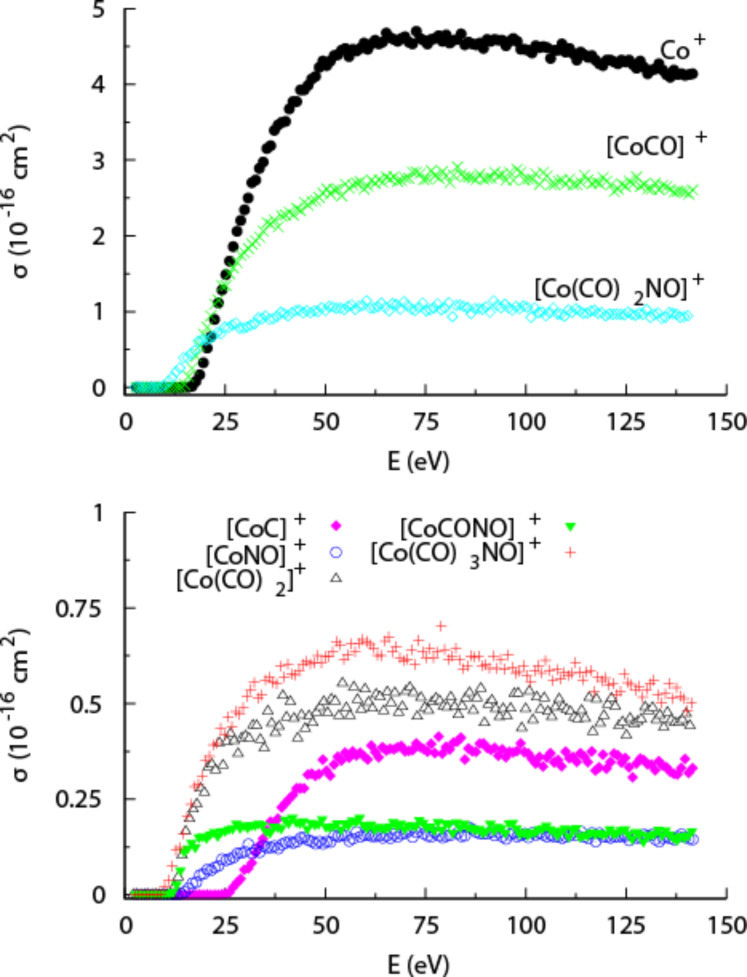
Energy dependence of the partial cross sections for positive ion fragments formed from Co(CO)_3_NO (reproduced with permission from [10], Copyright (2013) American Institute of Physics).

Figure 18 shows the absolute cross sections for the various fragments produced through DI of Co(CO)_3_NO as a function of the incident electron energy. At the maximum of the total cross section, around 50 eV, the most intense DI fragment is the bare cobalt cation (Co^+^) with a maximum absolute cross section of about 4.6 × 10^−16^ cm^2^, i.e., similar to that for a single CO loss through DEA. The second most efficient channel at this energy is the formation of [CoCO]^+^, with an absolute cross section of about 2.8 × 10^−16^ cm^2^. Hence, DI of Co(CO)_3_NO, unlike DEA, results largely in complete or almost complete dissociation of the precursor molecule. The relative cross sections for the loss of a single CO ligand or the NO group are nonetheless still appreciable, and at about 50 eV they amount to about 25% and 12.5% of that for the Co^+^ formation, respectively. Interestingly, the formation of [CoC]^+^ is also observed with a fairly high cross section above its formation threshold – about 3 × 10^−16^ cm^2^. All cross sections mentioned here are for incident energies of about 50 eV, where all channels have reached their maximum cross sections (Figure 18). At higher energies the cross-sections remain fairly constant. Conversely, the threshold for the individual channels is very different; for example, while the appearance energy for single CO loss is at about 8.47 ± 0.15 eV, that for Co^+^ formation is about 14.90 ± 0.15 eV [85]. The energy-dependent cross sections for these processes cross at about 25 eV and the single CO loss is thus the more efficient channel in the energy range from 8.47 ± 0.15 eV to about 25 eV (Figure 18).

**Figure 19:**
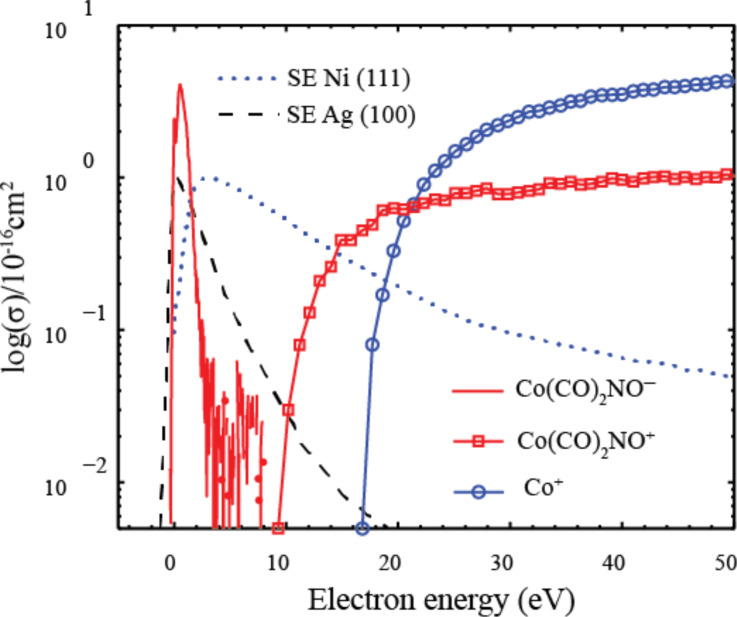
The partial cross sections for single CO loss through DEA (red solid line), for single CO loss through DI (red open squares), and for Co^+^ formation through DI (blue open circles), all adapted from Engmann et al. [10]. Also shown are the measured SE distributions from Ni(111) adapted from Schaefer et al. [6] (blue dotted line) and the measured SE distributions from Ag(100) adapted from Knights et al. [9] (black dashed line).

As discussed previously, to properly evaluate the efficiency of individual DEA and DI channels determined in gas phase studies in the context of FEBID, it is important to consider not only the energy-dependent reaction cross sections (as shown in Figure 17 and Figure 18), but also the energy distribution of the secondary electrons produced from the substrate and their overlap with the respective cross sections for each dissociation channel. This is a reflection of the fact that the overall efficiency of a given reaction pathway mediated by DEA, DI, or ND will be a convolution of the energy-dependent reaction cross section and the secondary electron yield at each energy. To demonstrate this, Figure 19 shows the partial cross section for single CO loss from Co(CO)_3_NO through both DEA and DI along with the cross section for the formation of Co^+^ through DI. On the same plot the measured SE distributions from Ni(111) and Ag(100) are also shown.

**Figure 20:**
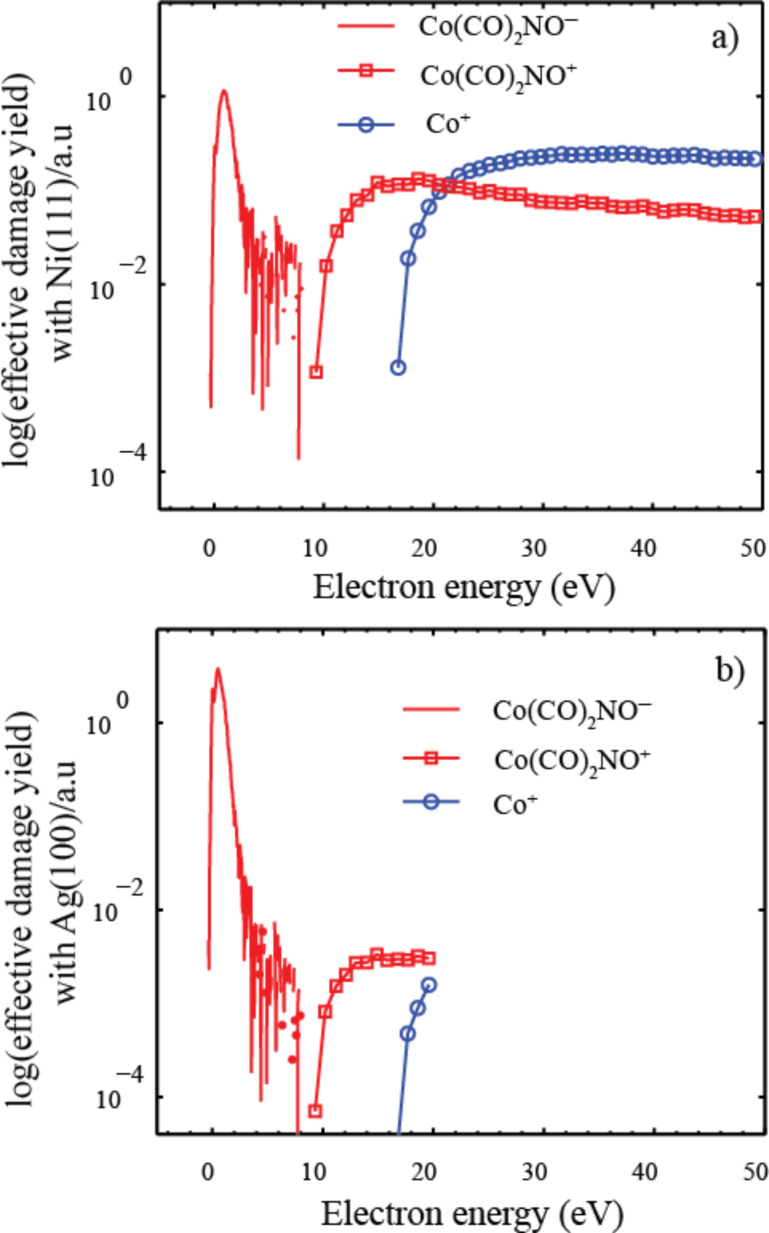
Predicted relative effective damage yield for single CO loss through DEA (red solid line), for single CO loss through DI (red open squares) and for the formation of Co^+^ through DI (blue open circles). Damage yields are derived from the product of the respective partial cross sections and the normalized measured SE yields for (a) Ni(111) and (b) Ag(100). The cross sections are adapted from [10] and the SE yields for Ni(111) and Ag(100) from [6] and [9], respectively.

Using the information contained in Figure 19, Figure 20a and Figure 20b show the predicted relative effective damage yield for each channel, derived from the product of the respective ion yields and the normalized measured SE yields for Ni(111) and Ag(100), respectively. It is clear from this comparison that the SE energy distribution will influence not only the relative importance of DEA compared to ND and DI, but may also cause the relative efficiency of individual DI channels to differ significantly from their relative cross sections in the gas phase. We recognize that the energy distribution of the SEs from the single crystal Ni(111) and Ag(100) surfaces is not likely to accurately reflect the SE energy distribution in a FEBID experiment where the substrate is polycrystalline or, as deposition proceeds, the deposit surface itself. The physisorbed precursor molecule and the background gas also play a role. Nevertheless, it is obvious from Figure 20b that a SE energy distribution similar to that for Ag(100) would strongly favor DEA over DI, and from the observed DI channels those with the lowest threshold energies would dominate, yielding a single CO loss rather than leading to Co^+^ formation. For the SE energy distribution measured for Ni(111) (Figure 20a), on the other hand, the integral damage yields through DEA and DI are comparable with Co^+^ formation being favored over single CO loss in DI.

Judging from the gas phase data alone, one would expect that if the decomposition of adsorbed Co(CO)_3_NO molecules is driven solely by DEA, it would mainly occur through CO desorption (see Figure 17) and would lead to a reduction of the relative Co/C/O ratio from 1:3:4 to about 1:2:3. The loss of nitrogen should be insignificant. In contrast, for a DI-driven decomposition process, NO desorption would be significant based on the integral cross sections (Figure 18) and the expected Co/N ratio remaining on the surface should be reduced from the initial 1:1 to about 1:0.25.

**Figure 21:**
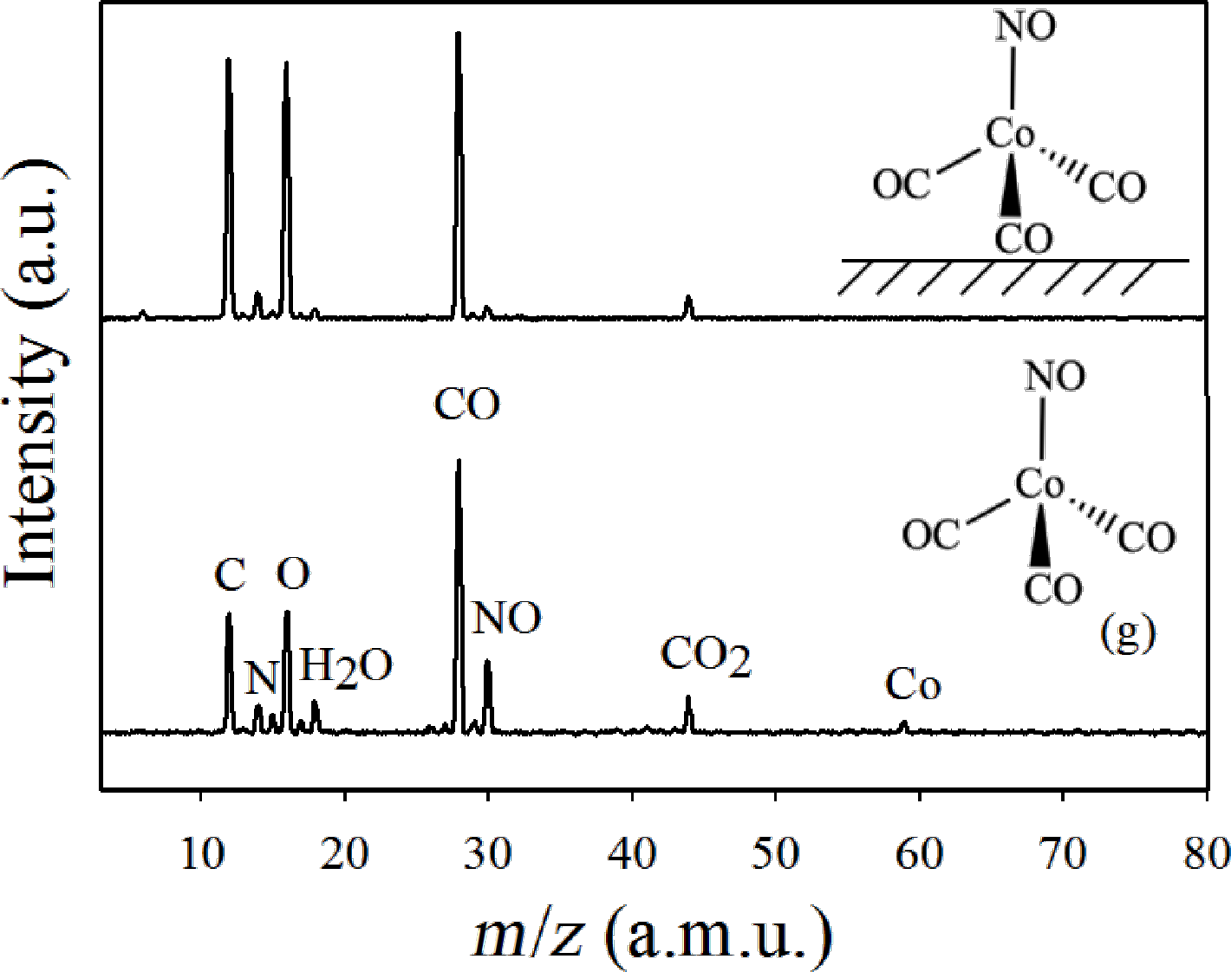
Positive ion (DI) mass spectra of (a) gas phase Co(CO)_3_NO, (b) volatile species desorbing from a 2.5 nm thick Co(CO)_3_NO film, adsorbed onto a gold substrate at 105 K, during irradiation with an electron dose of 5 × 10^16^ e^−^/cm^2^ at an electron energy of 500 eV (reproduced with permission from [25], Copyright (2013) American Chemical Society).

This can be compared with a UHV surface study of adsorbed Co(CO)_3_NO by Rosenberg et al. [25]. In this study the authors used XPS, MS, and RAIRS to examine metal–ligand bond dissociation caused by irradiation of the adsorbed precursor molecules with 500 eV primary electrons. Figure 21 shows mass spectra of (a) gas phase Co(CO)_3_NO and (b) the fragments desorbing during the electron irradiation of approximately 8–10 monolayers of Co(CO)_3_NO adsorbed on a polycrystalline Au surface. In the gas phase mass spectrum, the ratio of CO (*m*/*z* 28) to NO (*m*/*z* 30) is close to the stoichiometric composition of Co(CO)_3_NO. Conversely, the mass spectrum recorded during electron exposure of adsorbed Co(CO)_3_NO shows dominating CO desorption while the NO contribution is insignificant. This is observed for electron doses up to about 5 × 10^16^ e^−^/cm^2^, above which the CO desorption comes to a halt.

**Figure 22:**
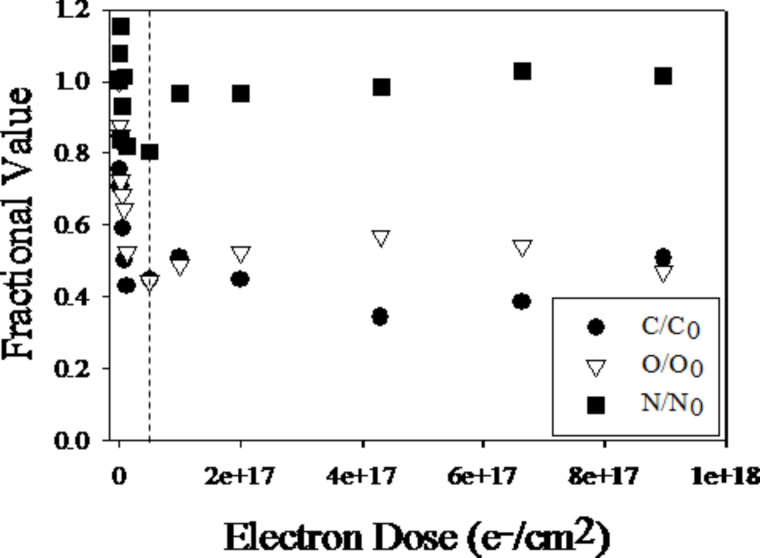
Electron dose dependence of the fractional coverage of carbon, oxygen and nitrogen from Co(CO)_3_NO adsorbed onto a gold substrate at 105 K and exposed to 500 eV electrons. The vertical dotted line represents an electron dose of 5 × 10^16^ e^−^/cm^2^ (reproduced with permission from [25], Copyright (2013) American Chemical Society).

The dominance of CO desorption from adsorbed Co(CO)_3_NO molecules exposed to electron irradiation is also reflected in the composition of the remaining deposit as measured with XPS [25]. Figure 22 shows the evolution of the fractional carbon, nitrogen, and oxygen content on the surface, referenced to the composition of the precursor prior to electron irradiation. While the fractional nitrogen content stays constant during the whole exposure time, the fractional oxygen and carbon contents fall to about 50% of their initial value by an electron dose of 5 × 10^16^ e^−^/cm^2^. Above 5 × 10^16^ e^−^/cm^2^, however, the fractional oxygen and carbon contents stay constant up to electron doses as high as 10^18^ e^−^/cm^2^. Although the fractional nitrogen content stays constant throughout the electron doses studied, it is reduced from the initial nitrosyl to a nitride species [25]. Reduction of the carbonyl carbon to graphitic carbon and concurrent conversion of the carbonyl and nitrosyl oxygen to an oxide species is also observed. While the nitride speciation change occurs at electron doses below 5 × 10^16^ e^−^/cm^2^ and is coincident with CO ejection, the carbon and oxygen changes primarily take place at higher electron doses. Furthermore, as a result of electron irradiation, changes in the cobalt region suggest the formation of a cobalt oxide and/or cobalt nitride.

The authors interpreted their data as a two-step process governing the electron-induced deposition of adsorbed Co(CO)_3_NO. The first (deposition) step occurs at low electron dose. In this step, one or more (an average of 1.5) CO ligands dissociate from the parent molecule and the NO ligand decomposes, producing a nitride species. The lack of NO desorption is also evident in the MS, which shows significant CO desorption from the surface. During this period, the cobalt is slightly oxidized and shows a peak broadening to a higher binding energy, likely resultant from oxide and/or nitride formation. This step is complete at an electron dose of about 5 × 10^16^ e^−^/cm^2^.

After this point, the second (decomposition) step can be observed. The partially decarbonylated species remaining; [(CO)_x_OCoN], undergoes an electron-stimulated decomposition of the remaining CO ligand(s) and adsorbed carbon is formed. More cobalt oxide is formed; likely due to reactions between reactive oxygen species released from the decomposition of the remaining CO ligands and Co atoms. The chemical composition of the final product formed due to electron irradiation of the physisorbed Co(CO)_3_NO film is a mixture of metallic cobalt, cobalt oxide and nitride, and adsorbed carbon. A similar evolution was observed when adsorbed Co(CO)_3_NO was exposed to X-ray radiation, indicating that the bulk of the decomposition is induced by SEs, rather than the 500 eV PEs [25].

Returning to the gas phase measurements of Co(CO)_3_NO, a decomposition process dominated by DEA would be expected to proceed through loss of a single CO ligand while DI would occur via a much more complete fragmentation. For an electron energy of 50 eV where the DI cross-sections reach their maximum values, a weighted average loss of slightly above 2 CO ligands is predicted for a DI process. The weighted average is estimated as the sum of the partial cross sections for the individual dissociation channels at 50 eV, multiplied by the number of CO ligands lost in each channel and divided by the total DI cross section at 50 eV. The average CO loss of about 1.5 observed in the surface experiments is therefore intermediate to what would be expected from a DEA- and DI-driven decomposition when considering the existing gas phase data alone. Cleavage of the N=O bond is neither observed in gas phase DEA, nor in DI. This is not surprising, as the BDE of nitric oxide is about 6.5 eV [37] and the activation barrier for the electron-induced formation of a nitride species from gaseous Co(CO)_3_NO is likely to be considerable. From comparison of surface and gas phase data it is thus likely that the decomposition observed at surfaces is initiated by a CO loss as proposed by Rosenberg et al. [25], though the gas phase experiments do not allow any clear conjecture on the underlying process (i.e., if the initial CO loss is through DEA or DI). The surface science studies, however, indicate that the decomposition of the unstable intermediate left after the initial CO loss proceeds through a surface-catalyzed conversion of the nitrogen from the nitrosyl group to the nitride species observed, which is consistent with CVD and FEBID from Co(CO)_3_NO showing the persistence of nitrogen in the deposits.

In summary for Co(CO)_3_NO, comparisons of currently available gas phase and surface science studies do not provide a definitive clear-cut answer as to the initial dissociation mechanism, although a combination from both DEA and DI channels seems most likely. However, even this assertion is speculative in the absence of any information on potential ND channels and a detailed analysis of the overlap between the individual DI and DEA channels and the actual SE energy distribution from the surface (which is currently not available).

Finally, it is worthwhile to take a brief look at gas phase and surface studies on the metal carbonyl compound W(CO)_6_. A recent DEA study of this compound by Wnorowski et al. [16] shows that, again, single CO loss is the most efficient channel and is confined to a fairly narrow energy region below 1 eV incident electron energy. Both the loss of two and three CO units, however, are fairly efficient through DEA and the integral ion yields for these channels are about 50% and 25% of that for a single CO loss, respectively. The loss of four CO units is also observed in DEA, but at higher energies (7–12 eV) and with low intensities (about 2.5% of that for single CO loss). No further fragmentation is observed in DEA to W(CO)_6_. Dissociative ionization of W(CO)_6_ is considerably more complex [17]. The loss of one CO (formation of [W(CO)_5_]^+^) has an appearance energy of about 10 eV and the appearance energy for the formation of W^+^ (loss of all ligands) is at about 20 eV. The intermediate fragments, [W(CO)*_n_*]^+^ (*n* = 1–4), appear at energies between these two fragments. Above 20 eV, the relative cross section for the formation of these cations stays fairly constant, with the formation of W^+^ (*n* = 0) as the most efficient channel and the relative cross section for the loss of one CO at about 50% of that for the W^+^ formation. The relative cross sections for the formation of other [W(CO)*_n_*]^+^ (*n* = 1–4) are, again, intermediate to these two. Above 20 eV, however, the formation of [(CO)*_n_*WC]^+^ (*n* = 0–3) appears, and at about 40 eV the efficiency of these channels is on the same order of magnitude as the respective [W(CO)*_n_*]^+^ channels. Further, both the doubly charged [W(CO)*_n_*]^2+^ and [(CO)*_n_*WC]^2+^ are also formed above 40 eV, though with efficiencies about an order of magnitude less than for their respective singly-charged species.

Despite the complexity of the DI fragmentation, a rough estimate of the DI average weighted CO loss of approximately 4 can be deduced from these ion yield curves at about 40 eV, where all single ionization channels are close to their maximum. This can be compared to an estimated DEA weighted average CO loss of 2. Thus, for a direct translation of the gas phase data to the surface experiments, considerably less CO loss would be expected for DEA-initiated deposition than for DI-initiated deposition; one would also expect considerable carbide formation via DI.

Similar surface experiments to those described in previous sections (500 eV PEs and Au surface at 160 K) have been conducted by Rosenberg et al. for W(CO)_6_ [26]. As expected, the mass spectrum of desorbed species upon electron irradiation shows CO as the dominating species. Further, the CO desorption decreases rapidly with increasing electron dose and above a dose of about 1 × 10^17^ e^−^/cm^2^ the CO desorption becomes insignificant. Consistent with these findings, XPS data reveals an average loss of 2 CO units for an electron dose of about 7 × 10^16^ e^−^/cm^2^. Above about 7 × 10^16^ e^−^/cm^2^, the dominant pathway becomes CO ligand decomposition rather than desorption, and the remaining W(CO)*_n_* is converted to graphitic carbon and a W(VI) oxide. The final deposits were found to consist of tungsten oxides encrusted in a carbonaceous matrix and no signs of carbide formation were observed.

In summary, the surface study revealed a deposition process for W(CO)_6_ that is similar to the other compounds examined here: a two-step process wherein the first is an electron-induced ligand loss and the second is characterized by decomposition of the remaining ligands. The number of ligands lost in the initial step is close to that observed in DEA rather than in DI and the absence of any carbide formation also favors DEA over DI. However, we stress again that ND is not included in these considerations, and the actual energy distribution of the secondary electrons is not taken into account when comparing the weighted average CO loss in the gas phase experiments.

## Conclusion

Here we have compared gas phase and surface data on low energy electron interaction with the common FEBID precursors MeCpPtMe_3_, Pt(PF_3_)_4_, Co(CO)_3_NO, and W(CO)_6_. For Pt(PF_3_)_4_ and MeCpPtMe_3_, single ligand loss dominates the initial step in their electron-induced decomposition at surfaces. This is also the most efficient DEA fragmentation channel in the gas phase, while DI predominantly leads to more complete fragmentation. Furthermore, in both cases single ligand loss through DEA in the gas phase is essentially exclusively confined to the electron energy range below 1 eV. Hence, an uncritical comparison between the current gas phase and surface data, as discussed here, indicates that the initial electron-induced fragmentation of these precursors is principally through DEA, and is primarily effected through secondary electrons with incident energies below 1 eV.

For the carbonylated precursors Co(CO)_3_NO and W(CO)_6_, deposition is somewhat different. For Co(CO)_3_NO, the surface studies show an initial average CO loss of about 1.5 ligands upon electron irradiation and essentially no NO loss. In gas phase DEA, the main channel is a single CO loss and the second most efficient channel is the loss of NO, with an absolute cross section that is about 10% of that for the single CO loss. The DEA-induced loss of two CO ligands has an absolute cross section that is about 5% of that for single CO loss. In gas phase DI above 50 eV, the loss of all ligands (formation of Co^+^) dominates and the weighted average CO loss above 50 eV is about 2. The weighted average NO loss in gas phase DI is about 0.7. It is therefore clear that, for Co(CO)_3_NO, neither the gas phase DEA nor the DI results correlate directly with the observed surface results. This is especially true in terms of accounting for the lack of nitrogen loss and the chemical transformation of the nitrogen species in the surface experiments. Indeed, the results obtained for Co(CO)_3_NO highlight the potentially important role that the surface can play in modifying the nature of the electron stimulated decomposition step.

For W(CO)_6_, the surface experiments show an initial CO ligand loss corresponding to an average of about 2. In gas phase DEA, single CO loss dominates below 1 eV, but the integral ion yield for the loss of two CO ligands is significant in the range of 2.5–4.5 eV and the loss of three CO ligands is also appreciable in the energy range of 3–6 eV. The weighted average CO loss through DEA in the gas phase estimated from the ion yield curves is about 2. Similarly to the other compounds, gas phase DI leads to much more extended fragmentation and an estimated average CO loss above about 40 eV is close to 4. Here, we find that the CO loss in the initial deposition step for W(CO)_6_ suggests DEA as the likely candidate; however, this is not conclusive. It is possible that for Co(CO)_3_NO and W(CO)_6_, deposition is a reflection of both DEA and DI processes.

It is unsurprising that low energy electron-induced decomposition of these organometallic compounds manifests differently when isolated in the gas phase and adsorbed on a surface. As previously mentioned, different relaxation processes are available at the surface and the lifetime of DEA resonances can be affected by polarization interactions with the surface. Furthermore, orientation effects may play a large role when molecules are adsorbed on surfaces – an effect that might explain the lack of NO desorption from Co(CO)_3_NO in the surface experiments. Additionally, the electron-induced loss of a single ligand from a precursor molecule may promote further ligand loss through surface interactions and/or chemical conversion of ligands (e.g., NO ligand in Co(CO)_3_NO), as is evident for all compounds compared here. This may be true even if such destabilization is not observed in the gas phase.

Furthermore, the current comparison is solely based on DEA and DI data from gas phase experiments and no systematic studies on ND cross sections and branching ratios exist. As has been demonstrated for Pt(PF_3_)_4_, the electronically inelastic cross sections can be very high and, in the gas phase, these are likely to relax predominantly through dissociation. Considerable progress is currently being made through quantum mechanical calculations on Pt(PF_3_)_4_, describing the potential energy surfaces for such electronically excited states [44]. These confirm the repulsive nature of the calculated states along a Pt–PF_3_ bond, indicating at least an initial single ligand loss. The resulting Pt(PF_3_)_3_ fragment may nevertheless contain enough internal energy for more extensive fragmentation, and thus, without further information on the branching ratios and cross sections for ND, a comparison between gas phase and surface data at this time remains incomplete.

Another major issue is that in the current surface experiments the adsorbed precursor molecules are exposed to electrons with fairly high energy, around 500 eV. The precursor molecules are thereby subjected to interaction with secondary electrons with a broad energy distribution and the energy dependence of individual processes are not manifested on the surface. Rather, these experiments reflect the cross sections for all processes (at the surface) convoluted with the SE energy distribution. This significantly complicates the direct comparison between gas phase and surface science studies.

To address these points, new experimental approaches are needed, most noticeably: a) ones that allow for the exploration of branching ratios and absolute cross sections for ND channels, which are perhaps achievable through a combination of the current crossed electron/molecule beam experiment with post-ionization sources; b) more detailed information on the secondary electron yield from surfaces exposed to electrons, including the surfaces that form as FEBID structures begin to grow; and c) experiments that allow electron energy-dependent studies on deposit formation and ligand desorption from adsorbed precursor molecules at lower energies, that includes the regime from about 0–15 eV (there are already a few laboratories with such setups, but to date their focus has been on other research topics). Moreover, the energy resolution of these electron sources must be sufficient to allow individual DEA resonances to be resolved, while the energy range must also be sufficient to scan through the onset region of DI and ND up to the maximum efficiency of these processes (typically in the regime between 50–70 eV). Our current inability to predict which precursors will react through which dissociation channels also highlights the need for a better theoretical understanding of electron/molecule interactions.

Despite the uncertainties associated with the current comparison, the comparison of gas phase and surface experiments on potential FEBID precursors is clearly an important step on the way to better understanding their initial decomposition mechanisms, illuminating the first step in the deposit formation in FEBID. For example, comparison between gas phase and surface studies has clearly implicated DEA rather than DI as the mechanism responsible for the initial decomposition of two of the precursors studied (MeCpPtMe_3_and Pt(PF_3_)_4_). This is the kind of information which in turn may aid the targeted design of precursors, whose initial decomposition step promotes further decomposition, and the control of which may thus be essential to optimize their performance.
